# Current status, trends, and predictions in the burden of silicosis in 204 countries and territories from 1990 to 2019

**DOI:** 10.3389/fpubh.2023.1216924

**Published:** 2023-07-13

**Authors:** Xinglin Yi, Yi He, Yu Zhang, Qiuyue Luo, Caixia Deng, Guihua Tang, Jiongye Zhang, Xiangdong Zhou, Hu Luo

**Affiliations:** ^1^Department of Respiratory Medicine, Southwest Hospital of Third Military Medical University, Chongqing, China; ^2^Department of Cardiovascular Medicine Department, Southwest Hospital of Third Military Medical University, Chongqing, China

**Keywords:** silicosis, age-standardized rate, jointpoint regression, prediction, global burden of disease

## Abstract

**Background:**

Silicosis, a severe lung disease caused by inhaling silica dust, predominantly affects workers in industries such as mining and construction, leading to a significant global public health challenge. The purpose of this study is to analyze the current disease burden of silicosis and to predict the development trend of silicosis in the future the world by extracting data from the GBD database.

**Methods:**

We extracted and analyzed silicosis prevalence, incidence, mortality, and disability-adjusted life years (DALYs) data from the Global Burden of Disease 2019 program for 204 countries and territories from 1990 to 2019. The association between the Sociodemographic Index (SDI) and the burden of age-standardized rates (ASRs) of DALYs has been examined at the regional level. Jointpoint regression analysis has been also performed to evaluate global burden trends of silicosis from 1990 to 2019. Furthermore, Nordpred age-period-cohort analysis has also been projected to predict future the burden of silicosis from 2019 to 2044.

**Results:**

In 2019, global ASRs for silicosis prevalence, incidence, mortality, and DALYs were 5.383, 1.650, 0.161, and 7.872%, respectively which are lower than that in 1990. The populations of 45–59 age group were more susceptible to silicosis, while those aged 80 or above suffered from higher mortality and DALY risks. In 2019, the most impacted nations by the burden of silicosis included China, the Democratic People’s Republic of Korea, and Chile. From 1990 to 2019, most regions observed a declining burden of silicosis. An “M” shaped association between SDI and ASRs of DALYs for silicosis was observed from 1990 to 2019. The age-period-cohort analysis forecasted a decreasing trend of the burden of silicosis from 2019 to 2044.

**Conclusion:**

Despite the overall decline in the global silicosis burden from 1990 to 2019, some regions witnessed a notable burden of this disease, emphasizing the importance of targeted interventions. Our results may provide a reference for the subsequent development of appropriate management strategies.

## 1. Introduction

Silicosis has been considered as an irreversible, progressive lung disease which is resulted from the inhalation of crystalline silica dust, a ubiquitous mineral present in various natural and industrial materials, such as sand, rock, artificial stone and quartz (1, 2). The debilitating condition primarily affects workers in industries including mining, construction, foundries, and ceramics, where exposure to silica dust is commonplace. As a debilitating disease, silicosis can lead to severe respiratory impairment, reduced quality of life and elevated risk of development of immune-related diseases such as tuberculosis and lung cancer (3). On the other hand, these health issues impose considerable strain on healthcare system, hinder workforce productivity, and generate substantial economic burden.

Recent advances in epidemiological research have shed light on the complex interplay of factors that contribute to the burden of silicosis, including variations in exposure levels, occupational practices, and individual susceptibility (4). The increasing recognition of silicosis as a global health challenge has spurred a demand for improved insight into its distribution and determinants across various countries and settings. This broader perspective is essential for identification of gaps in current prevention strategies, elimination of disparities in the burden of silicosis, and promotion of more effective and equitable interventions. In this context, a comprehensive assessment of the global and national burden of silicosis is crucial for evidence-based policy-making and prioritizing resources toward the most urgent public health requirements. By examining the trends in incidence, prevalence, mortality, and disability-adjusted life years associated with silicosis, researchers can develop a more thorough understanding of the disease’s dynamics and its impact on global and national health. The Global Burden of Diseases (GBD) database comprehensively evaluates published, publicly accessible, and contributed data on the burdens of prevalence, incidence and mortality of various causes and injuries enabling researchers to track temporal trends and analyze the disease burden over time. Using this database, Shi and colleagues demonstrated a decreasing incidence trend from 1990 to 2017 in most regions for the prevalence of malaria (5) Chen et al. have identified an overall decreased trend, from 1990 to 2019, of global burden for both mortality and disability-adjusted life years (DALYs) of silicosis and found an elevated mortality and DALYs rates for silicosis in such low- or middle- income countries as Portugal, China and Chile. However, the prevalent and incident trends of silicosis in more recent periods remain unclear and need further analysis (6).

In the present study, we aimed to evaluate the global, territorial and national trends in silicosis-related disease burden from 1990 to 2019 thoroughly by utilizing data from the GBD. Additionally, we employed age-period-cohort analysis to generate projections of future disease patterns.

## 2. Methods

### 2.1. Data source

The GBD database serves as a comprehensive and robust source of data for evaluating the health impacts of various diseases, injuries, and risk factors across the globe (7). The GBD project, coordinated by the Institute for Health Metrics and Evaluation (IHME), strives to systematically assess the descriptive epidemiology of a wide range of health conditions and their associated risk factors in multiple countries and regions (8). Comprehensive data regarding the GBD 2019 study can be accessed on the IHME.^1^

By compiling information from diverse sources such as systematic reviews, survey data, hospital administrative data, reported claims-based data and international organizations like the World Health Organization (WHO), the GBD database offers a wealth of information for public health researchers and policymakers. Utilizing DisMod-MR 2.0 statistical methods, the GBD database adjusted potential biases and inconsistencies in data, allowing for the generation of considerable and reliable imputed estimates of disease burden across different locations, time periods, and age groups (9).

In the GBD 2019, silicosis is identified as a chronic lung disease triggered by sustained inhalation of silica-laden dust particles. Within this study, a secondary analysis of the GBD 2019 dataset was conducted, centering on the disease burden imposed by silicosis across multiple countries and territories. Key metrics such as incidence, prevalence, mortality, and DALYs related to silicosis globally, and in distinct territories and nations from 1990 to 2019 were extracted. Additionally, demographic data like patient age and the Sociodemographic Index (SDI) were obtained for a more nuanced examination of the relationship between the burden of silicosis and these factors. Informed consent was not necessary, as individual-level primary data were not included in the analysis.

### 2.2. Data analyses

Incidence is determined by dividing the number of new cases by the overall population, while prevalence is gauged by dividing all cases by the overall population. Mortality rate is obtained by dividing the anticipated number of deaths by the overall population. DALYs are calculated by combining years of life lost (YLLs) – acquired by multiplying the anticipated number of deaths, participant age, and standard life expectancy for the relevant age – and years lived with disability (YLDs) – derived by multiplying prevalence and a distinct disability weight in the Bayesian regression model (6). All metrics of interest are displayed per 100,000. Age-standardized rates (ASRs) account for variations in age distribution among populations by applying the observed age-specific rates for each population to a standard population.

In this investigation, we collected and reported numerical data, ASRs and percentage change for estimated incidence, prevalence, mortality and DALYs across years (1990–2019) and age groups (5-year intervals) at levels of globe, territories and nations. We also examined the association between SDI which denotes the sociodemographic status and the burden of ASRs of DALYs at the regional level.

Jointpoint regression analysis was employed to gauge temporal trends in ASRs of estimated incidence, prevalence, mortality and DALYs. The annual percentage change (APC) and average annual percentage change (AAPC) were calculated using the Monte Carlo permutation test (10), along with its 95% confidence intervals (CIs). AAPCs with an *α* = 0.05 level are deemed significantly distinct from 0. A constant trend is indicated if 0 is encompassed within the 95% CIs, while increasing or decreasing trends are denoted if both limits of the 95% CIs are positive or negative, respectively.

Furthermore, we projected future trends in incidence, prevalence, DALYs, and mortality associated with silicosis which is grounded on a Nordpred age-period-cohort analysis that was implemented in R software (version 4.2.2) via the EasyGBDR package. It is a log-linear age–period–cohort model designed to predict the number or rate of new cases, which can moderate exponential growth and constrain linear trend projection to fit the recent trends, showing effective in predicting the future burden trends (11, 12). It extrapolates the most recent 5-year observed periods (three or four, depending on data availability) using a power function to temper growth. The recent decade’s linear trend is then adjusted in the second, third, and fourth prediction periods, either attenuating or accentuating it by 25, 50, and 75%, respectively. The method then calculates predictions for the ASRs of new cases in 2044 by averaging the projected incidence rates for the final two prediction periods, centered on 2044. All data analyses were executed using the open-source software R (version 4.2.2).

## 3. Results

### 3.1. Current status on global and regional levels

As delineated in Table 1, global ASRs per 100,000 population for silicosis in 2019 were as follows: prevalence, 5.383 (95% Uncertainty interval [UI]: 4.236 to 6.837); incidence, 1.650 (95% UI: 1.360 to 1.980); mortality, 0.161 (95% UI: 0.136 to 0.203); and DALYs, 7.872 (95% UI: 6.246 to 9.959). Supplementary Table S1 disclosed the aggregate global counts in 2019: prevalence, 2,648,972.875 (95% UI: 2,178,324.861 to 3,179,349.169); incidence, 138,971.030 (95% UI: 113,564.476 to 167,464.972); mortality, 12,886.686 (95% UI: 10,826.977 to 16,160.924); and DALYs, 655,762.889 (95% UI: 519,296.986 to 828,025.128). In 2019, East Asia emerged as the most affected region, accounting for approximately 90% of the total number of silicosis cases.

**Table 1:** ASRs of Prevalence, incidence, mortality, and DALYs for silicosis in various territories, 2019 (per 100,000 Population).

location	Prevalence (95% UI)	Incidence (95% UI)	Mortality (95% UI)	DALYs (95% UI)
Global	5.383 (4.236, 6.837)	1.650 (1.360, 1.980)	0.161 (0.136, 0.203)	7.872 (6.246, 9.959)
Andean Latin America	7.546 (6.098, 9.382)	0.075 (0.055, 0.109)	0.037 (0.024, 0.059)	1.055 (0.712, 1.599)
Australasia	1.260 (0.907, 1.859)	0.150 (0.114, 0.193)	0.025 (0.015, 0.081)	0.599 (0.408, 1.429)
Caribbean	0.046 (0.031, 0.072)	0.319 (0.256, 0.402)	0.013 (0.007, 0.023)	0.313 (0.171, 0.563)
Central Asia	31.600 (26.065, 37.872)	0.284 (0.210, 0.372)	0.005 (0.004, 0.007)	0.213 (0.153, 0.294)
Central Europe	5.342 (4.141, 6.845)	0.306 (0.238, 0.388)	0.039 (0.033, 0.050)	1.576 (1.252, 2.008)
Central Latin America	1.592 (1.212, 2.088)	0.450 (0.359, 0.560)	0.058 (0.048, 0.069)	2.294 (1.834, 2.828)
Central Sub-Saharan Africa	1.636 (1.229, 2.110)	0.140 (0.107, 0.182)	0.086 (0.018, 0.193)	2.118 (0.544, 4.568)
East Asia	110.240 (90.448, 133.420)	5.780 (4.751, 6.908)	0.406 (0.317, 0.556)	24.610 (18.650, 31.852)
Eastern Europe	5.126 (3.793, 6.812)	0.065 (0.048, 0.090)	0.011 (0.009, 0.016)	0.971 (0.671, 1.411)
Eastern Sub-Saharan Africa	1.226 (0.900, 1.763)	0.557 (0.442, 0.694)	0.074 (0.016, 0.144)	1.880 (0.560, 3.493)
High-income Asia Pacific	0.897 (0.657, 1.233)	0.608 (0.467, 0.798)	0.076 (0.053, 0.150)	1.807 (1.371, 2.884)
High-income North America	0.948 (0.742, 1.210)	0.069 (0.053, 0.089)	0.020 (0.017, 0.034)	0.534 (0.451, 0.740)
North Africa and Middle East	0.262 (0.190, 0.365)	0.220 (0.179, 0.273)	0.019 (0.010, 0.026)	0.494 (0.290, 0.693)
Oceania	5.294 (3.925, 6.918)	0.638 (0.502, 0.819)	0.059 (0.017, 0.125)	1.699 (0.923, 2.831)
South Asia	12.389 (9.473, 16.498)	0.022 (0.016, 0.031)	0.134 (0.060, 0.198)	3.433 (1.784, 4.880)
Southeast Asia	4.071 (3.006, 5.488)	0.238 (0.176, 0.315)	0.005 (0.002, 0.008)	0.706 (0.469, 1.012)
Southern Latin America	6.916 (5.468, 8.777)	0.333 (0.251, 0.431)	0.166 (0.140, 0.202)	3.879 (3.325, 4.516)
Southern Sub-Saharan Africa	5.906 (4.597, 7.447)	0.418 (0.332, 0.518)	0.094 (0.064, 0.117)	3.046 (2.361, 3.722)
Tropical Latin America	0.248 (0.179, 0.343)	0.004 (0.003, 0.006)	0.120 (0.109, 0.135)	4.913 (4.174, 5.872)
Western Europe	2.532 (2.070, 3.119)	0.143 (0.101, 0.219)	0.131 (0.112, 0.172)	2.192 (1.899, 2.795)
Western Sub-Saharan Africa	4.762 (3.848, 5.995)	0.019 (0.014, 0.026)	0.012 (0.006, 0.017)	0.379 (0.226, 0.575)

95% UI, 95% uncertainty intervals; ASRs, age-standardized rates, DALYs, disability-adjusted life years.

In the regional analysis for 2019, the three regions with the highest prevalence of silicosis were all located in Asia. Ranked by ASRs, East Asia topped (110.240, 95% UI: 90.448 to 133.420), followed by Central Asia (31.600, 95% UI: 26.065 to 37.872), and South Asia (12.389, 95%UI: 9.473 to 16.498). East Asia also recorded the highest ASRs for incidence (5.780, 95% UI: 4.751 to 6.908), mortality (0.406, 95% UI: 0.317 to 0.556), and DALYs (24.610, 95% UI: 18.65 to 31.852) of silicosis. Notably, the 95% lower UIs for prevalence, incidence, mortality, and DALYs in East Asia surpassed the 95% upper UIs for these measures in all other regions. This finding underscores a significantly elevated burden of silicosis in East Asia relative to other geographical regions.

**Figure 1:**
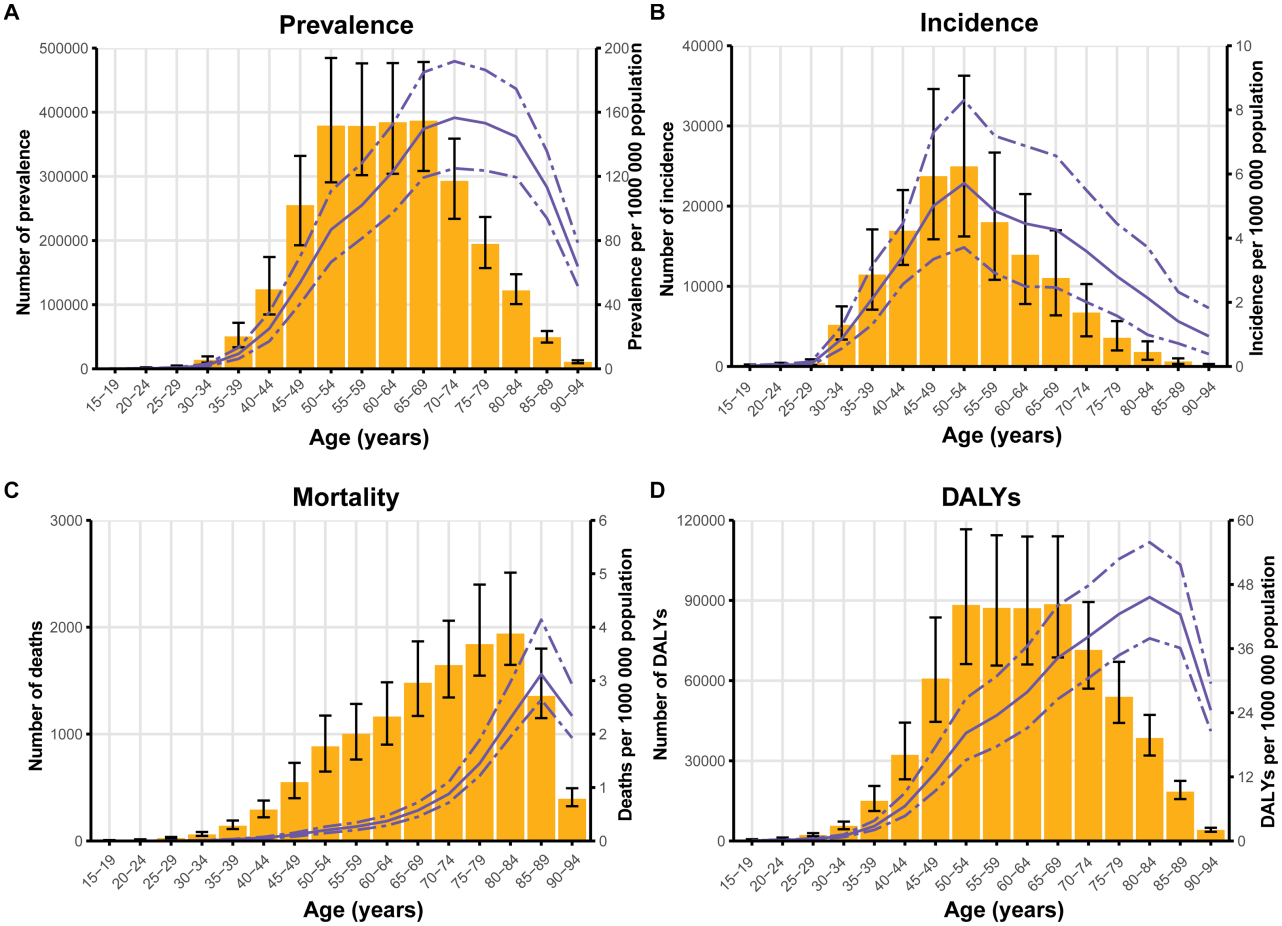
The numbers and ASRs of silicosis by age group for prevalence **(A)**, incidence **(B)**, mortality **(C)**, and DALYs **(D)** per 100,000 population in 2019. The yellow columns represent the numbers, while the purple lines reflect the ASRs. The error bar and dash-to-dash gap present the 95% UIs. ASRs, age-standardized rates; DALYs, disability-adjusted life years; UIs, uncertainty intervals.

Figure 1 displayed the numbers and ASRs for the global burden of silicosis by age group (5 years interval). The ASRs of prevalence for silicosis reached its peach at 70–74 years with 156.569 (95%UI: 125 to 191.777) per 100,100 population while ASRs of incidence peaked at 85–89 years with 5.713 (95%UI: 3.713 to 8.302) per 100,100 population. The individuals of 85–89 years had the highest ASRs of mortality for silicosis with 45.618 (95%UI: 37.903–55.885) while those of 85–89 years had the largest ASRs of DALYs with 3.121 (95%UI: 2.643–4.140) per 100,100 population.

### 3.2. Current status on a national level

**Figure 2:**
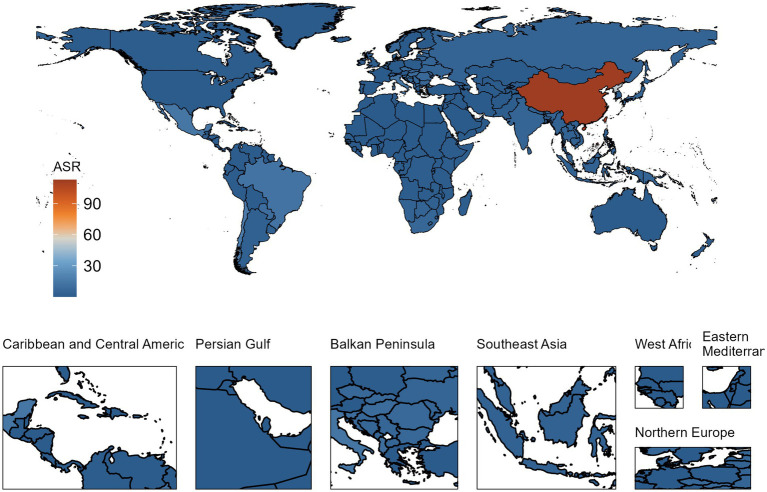
ASRs of prevalence for silicosis per 100,000 population in 2019.

In 2019, the nations most impacted by the burden of silicosis were China, the Democratic People’s Republic of Korea, and Chile. Regarding prevalence, China demonstrated the highest ASR of silicosis (113.149, 95% UI: 92.924 to 136.700, per 100,000 population), followed by the Democratic People’s Republic of Korea (51.190, 95% UI: 38.223 to 73.513), Chile (16.289, 95% UI: 12.792 to 20.858), Mexico (13.991, 95% UI: 11.225 to 17.544), and Italy (13.282, 95% UI: 10.781 to 16.497) (Supplementary Table S2; Figure 2).

In terms of incidence, China also exhibited the highest ASR (5.918, 95% UI: 4.866 to 7.073), with subsequent rankings held by the Democratic People’s Republic of Korea (3.150, 95% UI: 2.384 to 4.313), Chile (1.472, 95% UI: 1.147 to 1.889), Italy (1.194, 95% UI: 0.961 to 1.495), and Mexico (0.810, 95% UI: 0.642 to 1.018) (Supplementary Table S3).

Excluding Palau, which had a small population as well as mortality numbers in 2019, the Democratic People’s Republic of Korea presented the highest mortality ASRs for silicosis (0.599, 95% UI: 0.271 to 1.049), succeeded by Chile (0.511, 95% UI: 0.420 to 0.635), China (0.404, 95% UI: 0.312 to 0.564), Portugal (0.366, 95% UI: 0.305 to 0.439), and Taiwan (Province of China) (0.308, 95% UI: 0.213 to 0.415) (Supplementary Table S4).

**Figure 3:**
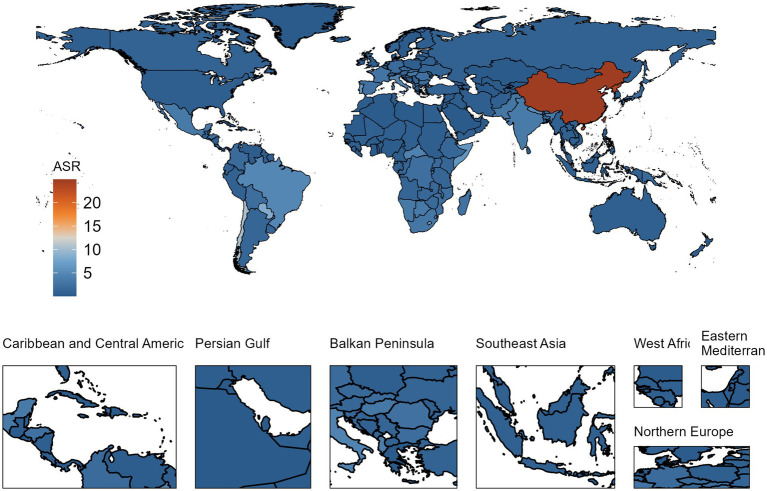
ASRs of DALYs for silicosis per 100,000 population in 2019.

When assessing the ASRs for DALYs, China (24.969, 95% UI: 18.900 to 32.513) and the Democratic People’s Republic of Korea (23.475, 95% UI: 13.458 to 36.796) displayed the highest rates, nearly identical to each other. Following these were Chile (10.424, 95% UI: 8.734 to 12.551), Paraguay (8.499, 95% UI: 5.666 to 11.602), Lesotho (7.650, 95% UI: 3.789 to 12.867), and Portugal (7.229, 95% UI: 5.998 to 8.770) (Supplementary Table S5, Figure 3).

### 3.3. Trends

An evaluation of longitudinal trends from 1990 to 2019, as presented in Table 2, denoted a global decline in ASRs for incidence, mortality, and DALYs. However, a definitive decrease in the ASR for prevalence remains indeterminate, with the 95% UI encompassing. As outlined in Table 2, the most substantial increases in the ASRs of silicosis prevalence from 1990 to 2019 were observed in Central Asia (1.610, 95% UI: 1.009 to 2.358, per 100,000 population), Southeast Asia (0.805, 95% UI: 0.542 to 1.163), and Southern Latin America (0.775, 95% UI: 0.544 to 1.026). In contrast, the most pronounced decreases were observed in Western Europe (−0.737, 95% UI: −0.796 to −0.676), High-income Asia Pacific (−0.728, 95% UI: −0.798 to −0.670), and High-income North America (−0.676, 95% UI: −0.739 to −0.596).

**Table 2:** Percentage changes in ASRs of prevalence, incidence, mortality, and DALYs for silicosis in various territories from 1990 to 2019 (per 100,000 population).

location	Prevalence (95% UI)	Incidence (95% UI)	Mortality (95% UI)	DALYs (95% UI)
Global	−0.046 (−0.178, 0.063)	−0.115 (−0.226, −0.010)	−0.589 (−0.679, −0.394)	−0.433 (−0.542, −0.271)
Andean Latin America	0.046 (−0.257, 0.260)	−0.021 (−0.321, 0.203)	−0.747 (−0.852, −0.468)	−0.727 (−0.832, −0.438)
Australasia	−0.200 (−0.364, 0.154)	−0.176 (−0.342, 0.244)	−0.682 (−0.823, 0.317)	−0.610 (−0.742, 0.107)
Caribbean	−0.073 (−0.182, 0.018)	−0.106 (−0.236, 0.015)	−0.420 (−0.576, −0.206)	−0.372 (−0.551, −0.130)
Central Asia	1.610 (1.009, 2.358)	1.168 (0.663, 1.723)	−0.452 (−0.599, −0.234)	−0.102 (−0.343, 0.204)
Central Europe	−0.406 (−0.500, −0.327)	−0.450 (−0.527, −0.393)	−0.876 (−0.898, −0.849)	−0.815 (−0.847, −0.772)
Central Latin America	−0.403 (−0.464, −0.332)	−0.467 (−0.514, −0.420)	−0.745 (−0.788, −0.698)	−0.649 (−0.695, −0.601)
Central Sub-Saharan Africa	0.075 (−0.057, 0.223)	0.109 (−0.028, 0.249)	−0.227 (−0.573, 0.444)	−0.204 (−0.531, 0.399)
East Asia	−0.096 (−0.222, 0.016)	−0.062 (−0.179, 0.040)	−0.603 (−0.732, −0.268)	−0.417 (−0.547, −0.209)
Eastern Europe	−0.032 (−0.166, 0.077)	−0.085 (−0.214, 0.018)	−0.675 (−0.828, −0.429)	−0.337 (−0.560, −0.119)
Eastern Sub-Saharan Africa	0.054 (0.000, 0.118)	0.027 (−0.024, 0.087)	−0.386 (−0.574, 0.021)	−0.368 (−0.583, 0.003)
High-income Asia Pacific	−0.728 (−0.798, −0.670)	−0.686 (−0.741, −0.612)	−0.779 (−0.837, −0.592)	−0.805 (−0.843, −0.709)
High-income North America	−0.676 (−0.739, −0.596)	−0.695 (−0.747, −0.626)	−0.623 (−0.678, −0.439)	−0.637 (−0.681, −0.545)
North Africa and Middle East	0.471 (0.263, 0.728)	0.571 (0.315, 0.861)	−0.501 (−0.690, −0.079)	−0.573 (−0.734, −0.288)
Oceania	0.500 (0.285, 0.747)	0.411 (0.236, 0.639)	−0.463 (−0.683, −0.123)	−0.265 (−0.489, 0.142)
South Asia	−0.182 (−0.234, −0.127)	−0.236 (−0.281, −0.194)	−0.566 (−0.686, 0.181)	−0.534 (−0.661, 0.016)
Southeast Asia	0.805 (0.542, 1.163)	0.758 (0.509, 1.118)	−0.571 (−0.726, −0.151)	0.155 (−0.103, 0.492)
Southern Latin America	0.775 (0.544, 1.026)	0.803 (0.594, 1.053)	−0.418 (−0.523, −0.276)	−0.397 (−0.496, −0.275)
Southern Sub-Saharan Africa	−0.030 (−0.134, 0.051)	−0.005 (−0.109, 0.074)	−0.279 (−0.499, −0.022)	−0.268 (−0.458, −0.067)
Tropical Latin America	−0.045 (−0.147, 0.052)	0.029 (−0.05, 0.119)	−0.247 (−0.324, −0.147)	−0.238 (−0.306, −0.155)
Western Europe	−0.737 (−0.796, −0.676)	−0.724 (−0.772, −0.653)	−0.715 (−0.756, −0.646)	−0.783 (−0.811, −0.738)
Western Sub-Saharan Africa	0.159 (0.042, 0.299)	0.175 (0.037, 0.327)	−0.364 (−0.573, 0.146)	−0.164 (−0.438, 0.419)

When examining incidence, the highest increases in ASRs were found in Central Asia (1.168, 95% UI: 0.663 to 1.723), Southern Latin America (0.803, 85% UI: 0.594 to 1.053), and Southeast Asia (0.758, 95% UI: 0.509 to 1.118). The largest decreases, however, were observed in Western Europe (−0.724, 95% UI: −0.772 to −0.653), High-income North America (−0.695, 95% UI: −0.747 to −0.626), and High-income Asia Pacific (−0.686, 95% UI: −0.741 to −0.612).

Most regions displayed absolute decreases (95% upper UI < 0.000) in ASRs of mortality from 1990 to 2019, with the most significant reductions observed in Central Europe (−0.876, 95% UI: −0.898 to −0.849), High-income Asia Pacific (−0.779, 95% UI: −0.837 to −0.592), and Andean Latin America (−0.747, −0.852 to −0.468). Similarly, an absolute decline in ASRs of DALYs from 1990 to 2019 was registered in the majority of regions. Central Europe (−0.815, −0.847 to −0.772), High-income Asia Pacific (−0.805, 95% UI: −0.843 to −0.709), and Western Europe (−0.783, 95% UI: −0.811 to −0.738) marked the most pronounced declines.

**Figure 4:**
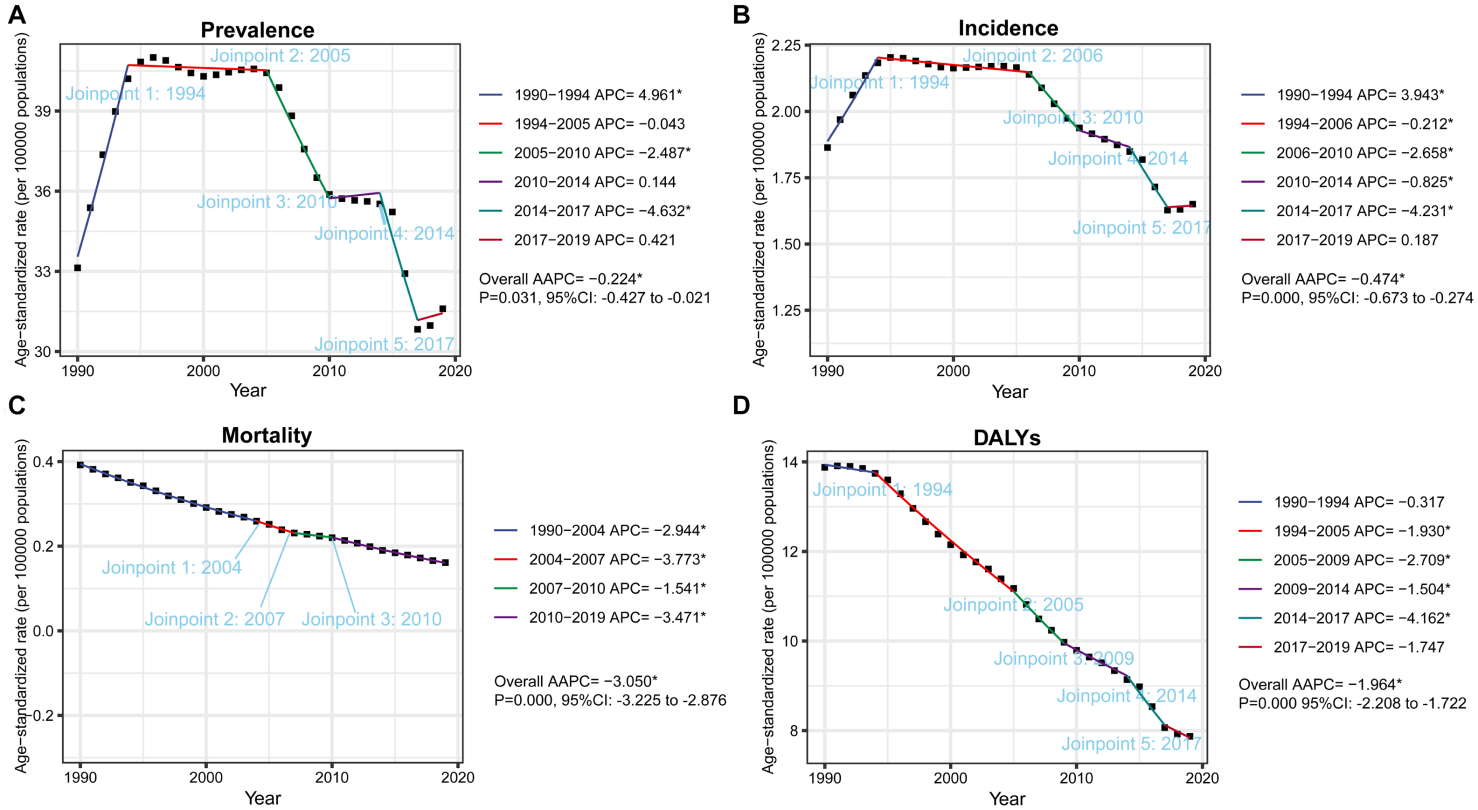
Global trends of ASRs, from 1990 to 2019, for prevalence **(A)**, incidence **(B)**, mortality **(C)** and DALYs **(D)** per 100,000 population of silicosis by Jointpoint Regression analysis. The symbol * represents statistical significance at *p* < 0.05. APC, annual percentage change, AAPC, average annual percentage change. CI, confidence interval.

As illustrated in Figure 4, Jointpoint regression analysis suggested inflection points and trends for the burden of silicosis from 1990 to 2019. The AAPCs of prevalence, incidence, mortality and DALYs were, respectively, −0.224% (95% CI: −0.427% to −0.021%, *p* = 0.031), −0.474% (95%CI: −0.673% to −0.274%, *p* = 0.000), −3.050% (95%CI: −3.225% to −2.876%, *p* = 0.000), and − 1.965% (95%CI: −2.208% to −1.722%, *p* = 0.000) which meant they all decreased in past 30 years. The prevalence rapidly increased before 1994 with APC at 4.961%, stabilized in 2005 with APC at −0.043%, then decreased of step-like to 2019 (Figure 4A). As for the trend of incidence of silicosis (Figure 4B), it rapidly increased before 1994 with APC at 3.943%, stabilized in 2006 with APC at −0.212%, then gradually decreased to 2019. As for trends of mortality (Figure 4C) and DALYs (Figure 4D), gradient decreases were observed from 1990 to 2019, with AAPC at −3.050% and − 1.964%, respectively.

### 3.4. Association between DALYs and SDI on a regional level

**Figure 5:**
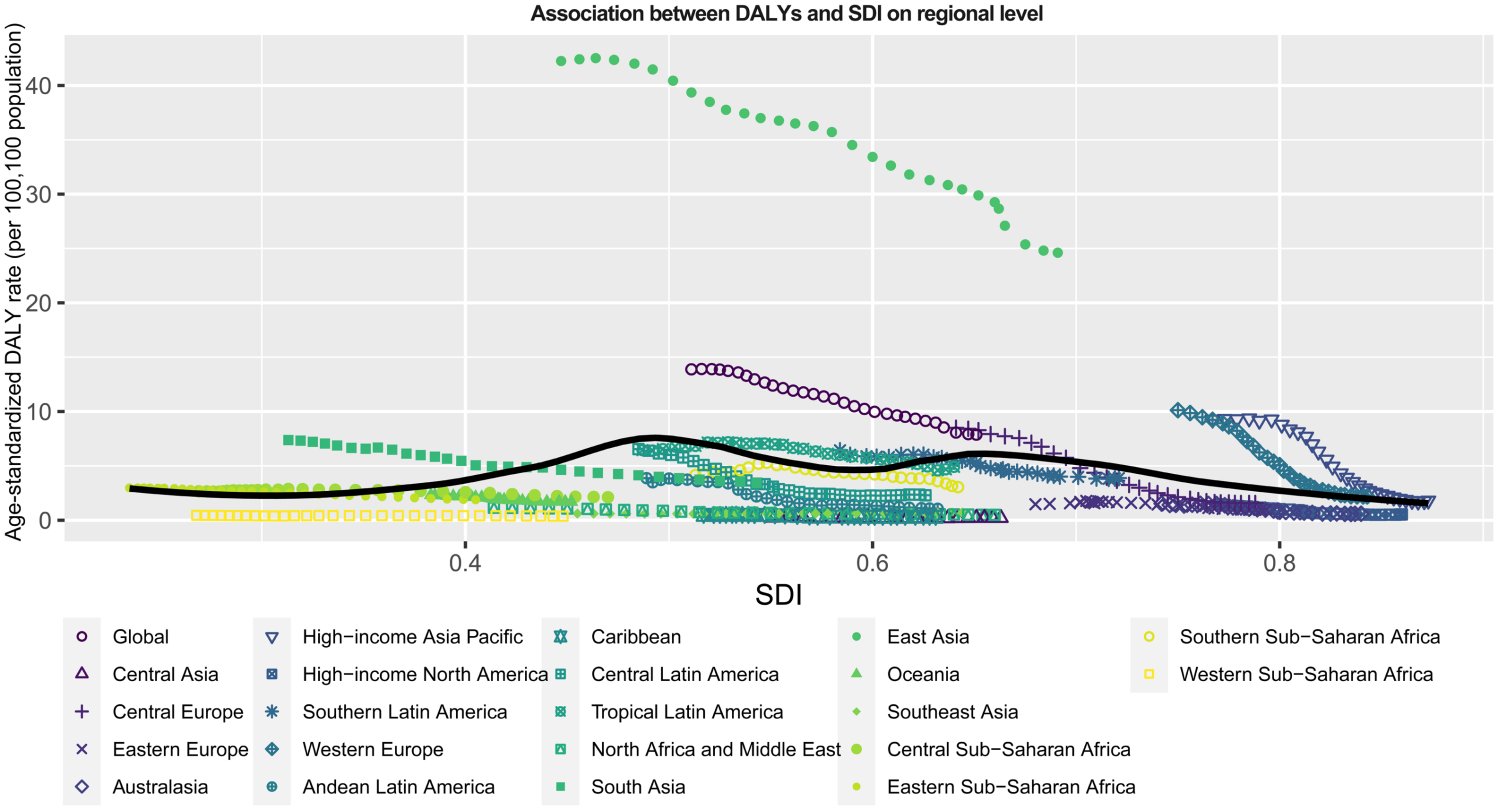
Association between SDI and ASRs of DALYs per 100,000 population for silicosis from 1990 to 2019 among 22 regions. The solid line portrays the estimated values, which have been computed by factoring in the sociodemographic index and disease rates across all locations. SDI, sociodemographic index.

As shown in Figure 5, an “M” shaped association between SDI and ASRs of DALYs for silicosis was observed from 1990 to 2019. With the growth of SDI up to about 0.5, the global burden of DALYs steadily increased. When SDI ranges from about 0.5 to 0.65, DALYs faced a slow decrease followed by a gradual increase. Then DALYs gradually decreased again with SDI at larger than 0.65. East Asia, Western Europe and High-income Asia Pacific had higher ASRs of DALYs for silicosis than expected DALYs rates, on the basis of SDI, from 1990 to 2019. On the contrary, Central Asia, Eastern Europe, Australia, High-income North America, Andean Latin America, Caribbean, Central Latin America, North Afica and Middle East, Oceania, Southeast Asia, Southern Sub-Saharan Africa and Western Sub-Saharan Africa had lower ASRs of DALYs for silicosis than expected DALYs rates, on the basis of SDI, from 1990 to 2019.

### 3.5. Predictions

**Figure 6:**
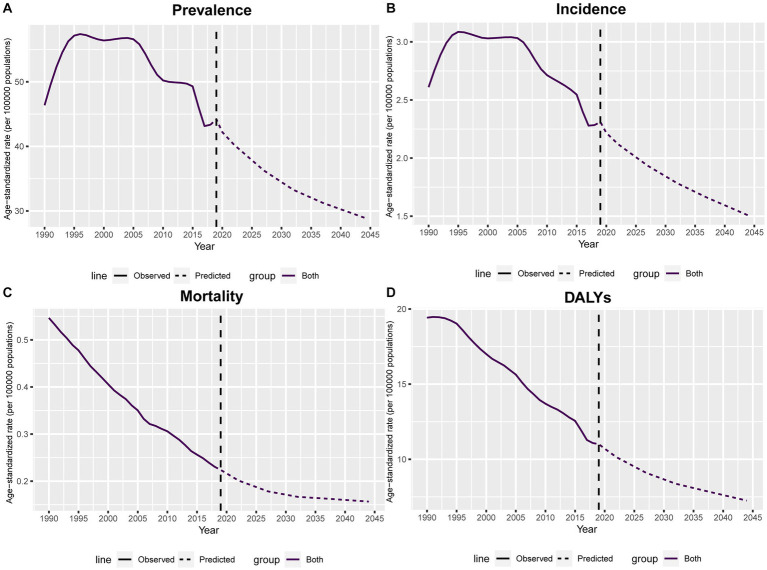
Nordpred based age-period-cohort prediction of global ASRs of prevalence **(A)**, incidence **(B)**, mortality **(C)** and DALYs **(D)** per 100,000 population for silicosis up to 2044. Solid lines indicate observed ASRs values while dashed lines denote the predicted.

Nordpred-based age-period-cohort analysis was performed to estimate trends of ASRs of prevalence, incidence, mortality and DALYs for silicosis up to 2044. As shown in Figure 6, the burden of silicosis gradually is going to step down from 2019 to 2044. During this period, ASRs of prevalence decreased by 10.904, ASRs of incidence decreased by 0.575, ASRs of mortality decreased by 0.048, and ASRs of DALYs decreased by 2.685. In 2044, global ASRs of prevalence, incidence, mortality and DALYs are 31.580, 1.650, 0.160, and 7.863, respectively.

## 4. Discussion

The present study provides an extensive examination of the current status and trends in silicosis and presents a detailed analysis of regional and national burdens, as well as the future burden associated with the disease. Our key findings were listed as follows: (1) In 2019, East Asia emerged as the most affected region of silicosis, accounting for approximately 90% of the total number of silicosis cases. China, the Democratic People’s Republic of Korea, Chile, and Mexico recorded the highest ASRs for both prevalent and incident cases of silicosis. They should warrant heightened attention and continued efforts toward prevention strategies. (2) Individuals aged 45–59 face a significantly greater risk of developing silicosis, while those aged 80 or above are at elevated risk of mortality and higher DALYs. These findings emphasize the importance of promoting the self-awareness among those in high-risk occupations who are over 45 years of age. (3) From 1990 to 2019, Central Asia, Southeast Asia, and the Southern Latin America regions experienced the most significant increases in ASRs of prevalence. Most regions exhibited decreases in the ASRs of mortality and DALYs. The most significant decreases have been observed in Central Europe, High-income Asia Pacific, and Western Europe. (4) Encouragingly, the global burden of silicosis declined significantly from 1990 to 2019 suggesting a continued decrease in the coming years.

The abovementioned findings indicated that the silicosis remains as important health problems in some countries and territories, though the global has attained great achievements in the prevention of silicosis. Evidence showed that the incident ASR remained relatively low over the past decades in high SDI countries. Moreover, in 2017, countries with an SDI greater than 0.7 registered a negative incident ASR of silicosis and presented negative correlation with increasing SDI (5). Our study has also identified similar findings that the incidence of silicosis witnessed a significant decrease in ASR of incidence in high-income regions in 2019, including high-income Asia Pacific, high-income North America, and Western Europe. Surprisingly, our study revealed that in 2019, a high burden of DALYs for silicosis was predominantly concentrated in countries with an SDI between 0.5 to 0.65, with a negative correlation observed between DALYs and SDI when it exceeds 0.65. This phenomenon may be attributed to the improved protection measures and increased awareness of self-protection among workers in the higher SDI countries.

Silicosis is prevalent among various industries including mining, quarrying, and construction. In China, the prevalence of silicosis among workers in the coal mining industry is alarmingly as high as 6%. Approximately 12.9% of cases were associated with the metallurgical industry, while 8.9% were linked to the production of construction materials (13). In India, a study published in 2016 reported that approximately 3 million miners in India at high risk of silicosis (14). Another study estimated that approximately 3 million individuals out of 8.5 million construction and building workers in India, are at risk of exposure to silica dust (15). An observatory study demonstrated that among the 729 silica-exposed individuals in India who underwent medical screening, 465 workers were diagnosed with silicosis with a median age of 45 years (16). The South African miner association pledged to eliminate silicosis, with the goal of no new cases by 2013. However, compared to a further study between 2004 and 2009 and a 1984 cohort of working gold miners, no substantial decrease was observed in the overall prevalence of silicosis.

Pulmonary tuberculosis (TB) is closely correlated with silicosis. Previous studies have proved that the likelihood of developing pulmonary TB increased significantly among artisanal and small-scale miners labour, contingent upon the levels of silica dust, exceeds that of healthy controls (17, 18) Moreover, TB infection is also positively related to the progression and severity of silicosis and stands as a primary contributor to morbidity and mortality among workers exposed to silica. As a result, regions with high background rates of TB and HIV can exhibit a notable prevalence of TB infection among workers exposed to silica. (19). Therefore, it is reasonable to infer that nations with high prevalence of TB may also face high burdens of silicosis, possibly explaining the higher rates of silicosis observed in countries like China and India (20).

According to a study published in 2022, global silica-related deaths and cases of silicosis were more prevalent in males in all age groups (6). In fact, the number of female silicosis patients was relatively small leading to a lower disease burden. Importantly, between 1990 and 2019, the disease burden of silicosis was demonstrated a general decline for both males and females, with a more pronounced decrease observed in males. According to the findings in the current study, individuals aged 65–79 are at a higher risk of developing silicosis, indicating that this disease is more chronic and often presents symptoms after several years. This is consistent with an occupational study conducted in 2019, in which Christopher and colleagues found a significantly higher proportion of silicosis among workers aged 65 or above in industries such as mining, foundry, and construction (21). It is suggested that these workers should be more vigilant about their health from a younger age, and doctors should pay closer attention to these workers to detect silicosis early.

Prompt detection and precise diagnosis of silicosis are of substantial importance which are primarily dependent on radiographic techniques, including X-rays, high-resolution computed tomography (HRCT), and pulmonary function assessments (22, 23). However, Radiologic features differ depending on the stage of diagnosis and have not been well described. A study on Chinese miners noted that despite finding pneumoconiosis on HRCT, 26.9% of people were negative on chest X-ray screening (24). Preliminary reports from Australian engineered stone workers showed that 43% patients were normal on chest X-ray, but silica-related disease was identified on CT, revealing a low sensitivity of chest X-ray in this circumstance (25). Therefore, HRCT should be highly recommended in early diagnosis of silicosis to facilitate earlier intervention.

Despite experiencing a period of rapid surge before 2005, the prevalence and incidence of global silicosis have gradually receded since then. Furthermore, this investigation is the first attempt to predict the future burden of silicosis based on the Nordpred age-period-cohort analysis. Surprisingly, the results forecast a progressive decline in ASRs of both prevalence and incidence globally prior to 2044. This is an encouraging outcome, because it provides evidence of the effectiveness of preventative measures for silicosis and supports the generalization of measures used in the most effective regions or countries. However, this investigation presents several limitations. Firstly, the exclusive utilization of GBD 2019 data introduces potential uncertainties within our estimations. Secondly, the GBD data underwent aggregation and adjustment through a back-estimation process derived from mortality data sources. However, it is important to note that the accuracy of the GBD data may be limited by the availability of data sources in certain regions. Thirdly, our analysis could not accurately assess the prevalence of silicosis obscured by coexisting pulmonary conditions, which may consequently lead to an underestimation of the true disease burden.

## 5. Conclusion

Despite the decline in global total burden over the past three decades, some regions continue to grapple with a high burden of silicosis in prevalence, incidence, mortality and DALYs. From 1990 to 2019, we observed an “M” shaped correlation between SDI and ASRs of DALYs for silicosis. Our study indicated a decrease in ASRs for prevalence, incidence, mortality, and DALYs from 2020 to 2044. These findings provide updated insights into the burden of silicosis for future research and highlight the requisite strategies and interventions necessary for effective prevention and management of the disease.

## Data availability statement

The original contributions presented in the study are included in the article/Supplementary material, further inquiries can be directed to the corresponding authors.

## Author contributions

XY and YH designed the study. GT and JZ completed the statistical analysis. YZ, QL, and CD drafted the manuscript. HL and XZ reviewed and edited the paper. All authors contributed to the article and approved the submitted version.

## Funding

This study was supported by the Chongqing Medical Scientific Research Project (No. 2020FYYX012).

## Conflict of interest

The authors declare that the research was conducted in the absence of any commercial or financial relationships that could be construed as a potential conflict of interest.

## Publisher’s note

All claims expressed in this article are solely those of the authors and do not necessarily represent those of their affiliated organizations, or those of the publisher, the editors and the reviewers. Any product that may be evaluated in this article, or claim that may be made by its manufacturer, is not guaranteed or endorsed by the publisher.

## Footnotes

^1^Website at: http://ghdx.healthdata.org/gbd-2019.
