# Supplementary material for: Current status, trends, and predictions in the burden of silicosis in 204 countries and territories from 1990 to 2019
Source: Front Public Health. 2023 Jul 13;11:1216924. doi: 10.3389/fpubh.2023.1216924 (PMC10372342; doi:10.3389/fpubh.2023.1216924)
Supplement: Supplementary file 1 [file Table_1.DOCX]

**Table S1.** Numbers of silicosis on a regional level in 2019 (per 100,000 Population)

| location | Prevalence (95% UI) | Incidence (95% UI) | Mortality (95% UI) | DALYs (95% UI) |
| --- | --- | --- | --- | --- |
| Global | 2648972.875 (2178324.861, 3179349.169) | 138971.030 (113564.476, 167464.972) | 12886.686 (10826.977, 16160.924) | 655762.889 (519296.986, 828025.128) |
| Andean Latin America | 687.748 (503.884, 989.825) | 43.884 (32.450, 63.451) | 20.469 (13.208, 32.952) | 603.186 (408.634, 914.680) |
| Australasia | 693.338 (499.531, 1041.358) | 75.873 (53.722, 116.430) | 13.459 (7.739, 44.903) | 307.809 (208.193, 738.226) |
| Caribbean | 23.677 (15.951, 36.912) | 2.006 (1.340, 3.297) | 6.725 (3.801, 11.943) | 159.577 (87.080, 285.727) |
| Central Asia | 642.309 (458.834, 876.460) | 56.144 (40.199, 78.925) | 2.419 (1.902, 3.735) | 147.643 (104.648, 206.501) |
| Central Europe | 10528.787 (8072.486, 13404.373) | 520.055 (399.778, 667.177) | 87.428 (72.706, 111.198) | 3218.664 (2571.526, 4059.493) |
| Central Latin America | 18147.227 (14680.838, 22580.938) | 1124.084 (900.621, 1400.702) | 132.862 (109.523, 158.771) | 5487.536 (4378.117, 6786.285) |
| Central Sub-Saharan Africa | 714.319 (530.971, 936.921) | 78.093 (58.558, 100.925) | 39.841 (7.156, 91.877) | 1157.497 (267.678, 2609.841) |
| East Asia | 2404151.523 (1969020.794, 2910635.921) | 122024.687 (99052.050, 147439.651) | 8060.214 (6223.443, 11061.622) | 529777.405 (402152.191, 687117.424) |
| Eastern Europe | 16463.898 (12092.751, 21880.923) | 808.187 (590.082, 1070.205) | 37.443 (29.759, 54.489) | 3140.560 (2189.868, 4577.617) |
| Eastern Sub-Saharan Africa | 2260.191 (1719.645, 2974.412) | 228.788 (174.466, 296.203) | 109.25 (22.662, 215.869) | 3165.866 (919.752, 6029.097) |
| High-income Asia Pacific | 28572.152 (22493.734, 36013.779) | 2602.097 (2048.649, 3229.893) | 435.879 (304.378, 861.842) | 9518.643 (7245.585, 15185.237) |
| High-income North America | 5873.204 (4564.666, 7627.789) | 405.718 (308.011, 538.917) | 129.63 (107.472, 223.683) | 3199.380 (2685.437, 4501.082) |
| North Africa and Middle East | 988.294 (708.705, 1360.689) | 89.955 (66.430, 125.997) | 77.449 (43.139, 110.997) | 2435.634 (1452.746, 3452.635) |
| Oceania | 384.509 (282.445, 512.763) | 29.398 (21.709, 38.268) | 2.616 (0.786, 5.636) | 115.764 (66.114, 183.665) |
| South Asia | 67446.569 (54165.787, 85441.703) | 4851.292 (3897.993, 6079.147) | 1718.229 (742.480, 2574.908) | 48275.940 (24804.612, 69386.064) |
| Southeast Asia | 26332.157 (19369.685, 35591.714) | 1691.835 (1240.209, 2249.215) | 27.332 (13.914, 47.141) | 4611.163 (3040.105, 6675.301) |
| Southern Latin America | 5973.442 (4718.305, 7592.255) | 535.378 (419.925, 687.673) | 142.263 (120.092, 173.042) | 3268.262 (2783.647, 3815.693) |
| Southern Sub-Saharan Africa | 3292.234 (2527.816, 4231.884) | 254.840 (199.824, 317.533) | 50.216 (34.476, 62.411) | 1800.557 (1398.958, 2199.600) |
| Tropical Latin America | 30876.839 (23626.410, 41276.824) | 1532.496 (1169.613, 2012.323) | 290.694 (264.931, 326.907) | 12277.320 (10403.886, 14721.922) |
| Western Europe | 24467.847 (19975.984, 30335.514) | 1978.038 (1565.872, 2485.094) | 1477.064 (1259.601, 1925.636) | 22095.266 (19093.418, 27902.862) |
| Western Sub-Saharan Africa | 452.612 (319.512, 629.053) | 38.183 (27.887, 52.465) | 25.204 (14.213, 39.757) | 999.217 (591.745, 1586.527) |
