# Supplementary material for: Current status, trends, and predictions in the burden of silicosis in 204 countries and territories from 1990 to 2019
Source: Front Public Health. 2023 Jul 13;11:1216924. doi: 10.3389/fpubh.2023.1216924 (PMC10372342; doi:10.3389/fpubh.2023.1216924)
Supplement: Supplementary file 2 [file Table_2.DOCX]

**Table S2.** Numbers and ASRs of prevalence for silicosis in nations in 2019 (per 100,000 Population)

| **location** | **Location** | **ASR s per 100 000 (95% UI)** |
| --- | --- | --- |
| China | 2384598.402 (1954213.818, 2881931.942) | 113.149 (92.924, 136.700) |
| Democratic People's Republic of Korea | 17156.868 (12864.021, 24715.677) | 51.190 (38.223, 73.513) |
| Chile | 3944.859 (3097.312, 5051.415) | 16.289 (12.792, 20.858) |
| Mexico | 16839.175 (13515.732, 21048.520) | 13.991 (11.225, 17.544) |
| Italy | 20443.595 (16521.796, 25497.801) | 13.282 (10.781, 16.497) |
| Brazil | 30336.345 (23182.581, 40642.114) | 12.469 (9.524, 16.639) |
| Palau | 2.609 (1.893, 3.429) | 11.227 (8.378, 14.461) |
| Albania | 430.758 (316.388, 616.072) | 10.251 (7.487, 14.464) |
| Slovenia | 366.574 (272.825, 481.912) | 10.046 (7.500, 13.129) |
| Paraguay | 540.493 (418.789, 693.834) | 9.147 (7.099, 11.668) |
| Hungary | 1600.635 (1230.009, 2066.264) | 9.004 (6.934, 11.753) |
| Slovakia | 666.945 (491.023, 889.013) | 7.511 (5.509, 9.986) |
| Romania | 2501.985 (1853.892, 3279.545) | 7.426 (5.538, 9.800) |
| Bulgaria | 913.354 (686.680, 1188.283) | 7.021 (5.376, 9.150) |
| Croatia | 528.677 (387.918, 687.640) | 7.010 (5.176, 9.157) |
| Czechia | 1324.521 (999.722, 1700.031) | 6.988 (5.242, 9.113) |
| South Africa | 2842.455 (2196.134, 3652.389) | 6.374 (4.963, 8.087) |
| Kiribati | 4.493 (3.156, 6.406) | 6.288 (4.555, 8.937) |
| Japan | 26605.823 (21051.782, 33330.975) | 6.228 (4.905, 7.878) |
| Norway | 521.990 (410.459, 675.118) | 6.163 (4.844, 8.030) |
| Latvia | 202.116 (149.550, 261.569) | 6.154 (4.496, 8.046) |
| Taiwan (Province of China) | 2396.253 (1964.968, 2944.910) | 6.006 (4.933, 7.352) |
| Serbia | 849.956 (610.495, 1120.752) | 5.883 (4.252, 7.840) |
| North Macedonia | 182.333 (132.885, 241.346) | 5.752 (4.224, 7.609) |
| Lesotho | 72.782 (53.375, 127.972) | 5.730 (4.258, 9.756) |
| Bosnia and Herzegovina | 314.864 (224.687, 427.832) | 5.604 (4.027, 7.551) |
| Montenegro | 51.784 (36.820, 69.252) | 5.500 (3.919, 7.320) |
| Ukraine | 3840.968 (2861.084, 5083.293) | 5.466 (4.038, 7.270) |
| Republic of Moldova | 303.207 (217.355, 414.398) | 5.454 (3.953, 7.442) |
| Estonia | 119.203 (86.517, 160.700) | 5.450 (3.941, 7.354) |
| Papua New Guinea | 263.879 (192.151, 350.065) | 5.423 (3.994, 7.124) |
| Belarus | 783.720 (577.069, 1027.582) | 5.245 (3.880, 6.904) |
| India | 60499.626 (48358.679, 76926.477) | 5.227 (4.220, 6.612) |
| Eswatini | 29.017 (21.116, 44.903) | 5.182 (3.860, 7.853) |
| Maldives | 17.773 (12.889, 24.094) | 5.161 (3.730, 6.907) |
| Northern Mariana Islands | 2.879 (2.036, 3.958) | 5.127 (3.725, 6.851) |
| Marshall Islands | 1.783 (1.300, 2.401) | 5.117 (3.796, 6.804) |
| Tokelau | 0.068 (0.049, 0.091) | 5.106 (3.715, 6.849) |
| Vanuatu | 8.854 (6.522, 12.020) | 5.098 (3.824, 6.862) |
| American Samoa | 2.458 (1.752, 3.312) | 5.044 (3.617, 6.780) |
| Guam | 9.686 (6.910, 13.016) | 5.040 (3.607, 6.734) |
| Cook Islands | 1.255 (0.899, 1.710) | 5.031 (3.617, 6.835) |
| Solomon Islands | 15.493 (11.186, 20.772) | 5.020 (3.740, 6.589) |
| Russian Federation | 10970.577 (8002.521, 14608.601) | 4.983 (3.653, 6.666) |
| Lithuania | 244.106 (183.501, 320.483) | 4.972 (3.699, 6.615) |
| Samoa | 7.268 (5.236, 9.783) | 4.896 (3.546, 6.515) |
| Botswana | 64.253 (45.186, 88.671) | 4.844 (3.466, 6.553) |
| Fiji | 37.700 (26.711, 51.059) | 4.823 (3.474, 6.469) |
| Malaysia | 1288.612 (915.570, 1715.886) | 4.682 (3.372, 6.198) |
| Tonga | 3.717 (2.716, 4.936) | 4.682 (3.420, 6.208) |
| Niue | 0.102 (0.073, 0.137) | 4.652 (3.336, 6.207) |
| Tuvalu | 0.478 (0.344, 0.652) | 4.636 (3.368, 6.219) |
| Nauru | 0.197 (0.137, 0.270) | 4.627 (3.405, 6.097) |
| Micronesia (Federated States of) | 3.431 (2.442, 4.621) | 4.606 (3.383, 6.140) |
| Timor-Leste | 37.014 (26.928, 49.156) | 4.487 (3.266, 5.921) |
| Thailand | 4629.738 (3392.461, 6281.375) | 4.442 (3.281, 6.002) |
| Seychelles | 5.082 (3.560, 6.949) | 4.345 (3.067, 5.908) |
| Namibia | 58.924 (43.024, 76.910) | 4.331 (3.189, 5.690) |
| Lao People's Democratic Republic | 199.829 (143.187, 269.548) | 4.283 (3.093, 5.784) |
| Mauritius | 76.579 (53.818, 104.420) | 4.273 (3.036, 5.809) |
| Sri Lanka | 1090.500 (769.771, 1490.332) | 4.178 (2.984, 5.699) |
| Indonesia | 9768.862 (7125.318, 13255.153) | 4.107 (3.022, 5.499) |
| Viet Nam | 3880.727 (2801.830, 5251.162) | 3.887 (2.835, 5.224) |
| Myanmar | 1860.941 (1347.658, 2500.963) | 3.804 (2.778, 5.068) |
| Cambodia | 464.159 (335.688, 622.577) | 3.680 (2.674, 4.880) |
| Philippines | 2977.843 (2177.515, 4032.756) | 3.527 (2.639, 4.784) |
| Zimbabwe | 224.802 (169.519, 295.294) | 3.368 (2.608, 4.319) |
| Bangladesh | 4144.915 (3118.243, 5373.698) | 3.327 (2.495, 4.279) |
| Argentina | 1843.413 (1394.797, 2471.020) | 3.293 (2.503, 4.407) |
| Uruguay | 184.869 (133.656, 251.748) | 3.183 (2.304, 4.335) |
| Nepal | 663.447 (507.897, 858.384) | 3.153 (2.425, 4.053) |
| Bhutan | 15.018 (11.673, 19.362) | 2.841 (2.203, 3.646) |
| New Zealand | 196.425 (142.421, 268.076) | 2.377 (1.733, 3.229) |
| Singapore | 157.555 (110.451, 220.453) | 2.200 (1.562, 3.055) |
| Republic of Korea | 1805.300 (1295.653, 2488.805) | 2.036 (1.467, 2.802) |
| Burundi | 76.153 (53.119, 104.770) | 2.016 (1.440, 2.706) |
| Madagascar | 171.697 (120.107, 241.371) | 1.995 (1.404, 2.731) |
| Pakistan | 2123.563 (1586.502, 2751.294) | 1.994 (1.531, 2.563) |
| Djibouti | 8.948 (6.418, 12.326) | 1.855 (1.349, 2.486) |
| United Kingdom | 2183.458 (1676.393, 2867.099) | 1.815 (1.398, 2.373) |
| Somalia | 99.440 (67.549, 146.899) | 1.765 (1.232, 2.564) |
| Brunei Darussalam | 3.475 (2.390, 4.947) | 1.764 (1.242, 2.492) |
| Kenya | 343.205 (264.671, 450.271) | 1.762 (1.366, 2.301) |
| Rwanda | 89.651 (62.430, 136.824) | 1.749 (1.221, 2.633) |
| Mozambique | 167.335 (117.131, 245.866) | 1.745 (1.225, 2.530) |
| Democratic Republic of the Congo | 497.057 (365.077, 650.927) | 1.682 (1.244, 2.193) |
| Armenia | 68.963 (48.304, 116.572) | 1.620 (1.148, 2.718) |
| Zambia | 89.441 (65.404, 119.953) | 1.620 (1.181, 2.155) |
| Malawi | 104.218 (74.165, 141.816) | 1.614 (1.153, 2.164) |
| South Sudan | 50.061 (38.103, 65.029) | 1.573 (1.201, 2.034) |
| Comoros | 7.017 (5.146, 9.243) | 1.568 (1.145, 2.065) |
| Angola | 139.970 (101.619, 186.917) | 1.566 (1.173, 2.062) |
| Central African Republic | 27.317 (19.914, 36.990) | 1.556 (1.172, 2.047) |
| Colombia | 804.156 (561.791, 1296.235) | 1.531 (1.069, 2.467) |
| Congo | 32.077 (24.152, 42.693) | 1.512 (1.151, 1.992) |
| Bolivia (Plurinational State of) | 132.723 (91.863, 221.524) | 1.511 (1.050, 2.552) |
| Uganda | 179.292 (130.742, 238.001) | 1.489 (1.076, 1.988) |
| United Republic of Tanzania | 309.673 (234.065, 410.617) | 1.435 (1.085, 1.891) |
| Equatorial Guinea | 5.619 (4.247, 7.533) | 1.419 (1.088, 1.879) |
| Ethiopia | 533.139 (413.443, 695.934) | 1.418 (1.099, 1.857) |
| Gabon | 12.279 (9.180, 16.521) | 1.327 (0.990, 1.783) |
| Eritrea | 29.115 (21.056, 39.143) | 1.325 (0.975, 1.767) |
| Canada | 871.708 (626.893, 1298.125) | 1.250 (0.907, 1.802) |
| Ecuador | 189.865 (137.056, 261.285) | 1.241 (0.907, 1.706) |
| Poland | 796.401 (666.909, 962.709) | 1.230 (1.025, 1.490) |
| Peru | 365.160 (256.340, 532.641) | 1.142 (0.797, 1.669) |
| Australia | 496.913 (345.408, 828.719) | 1.052 (0.730, 1.757) |
| Honduras | 59.362 (43.503, 80.648) | 1.017 (0.738, 1.401) |
| Tajikistan | 44.802 (31.318, 63.482) | 0.950 (0.673, 1.322) |
| Mongolia | 20.011 (13.726, 26.914) | 0.923 (0.640, 1.247) |
| United States of America | 5000.951 (3912.487, 6416.861) | 0.910 (0.715, 1.165) |
| Azerbaijan | 80.711 (56.112, 113.037) | 0.880 (0.623, 1.218) |
| Uzbekistan | 173.650 (115.123, 240.723) | 0.867 (0.607, 1.170) |
| Turkmenistan | 32.064 (22.107, 45.528) | 0.852 (0.598, 1.225) |
| Costa Rica | 43.169 (31.862, 58.925) | 0.850 (0.624, 1.157) |
| Kyrgyzstan | 37.083 (25.321, 52.229) | 0.836 (0.572, 1.192) |
| Georgia | 49.948 (35.206, 69.602) | 0.823 (0.583, 1.138) |
| Kazakhstan | 135.076 (90.816, 188.877) | 0.780 (0.537, 1.096) |
| Panama | 31.484 (22.155, 44.543) | 0.764 (0.537, 1.081) |
| Nicaragua | 32.613 (23.839, 44.433) | 0.760 (0.551, 1.052) |
| Venezuela (Bolivarian Republic of) | 215.077 (155.322, 299.353) | 0.757 (0.547, 1.058) |
| Guatemala | 80.264 (57.386, 109.385) | 0.719 (0.509, 0.990) |
| El Salvador | 41.925 (30.088, 58.179) | 0.701 (0.500, 0.975) |
| Greenland | 0.452 (0.319, 0.611) | 0.668 (0.483, 0.902) |
| Portugal | 135.219 (102.823, 177.048) | 0.536 (0.401, 0.715) |
| Iran (Islamic Republic of) | 262.694 (192.795, 357.539) | 0.399 (0.292, 0.548) |
| Turkey | 248.345 (167.148, 417.288) | 0.296 (0.199, 0.500) |
| United Arab Emirates | 8.797 (5.457, 13.323) | 0.284 (0.204, 0.387) |
| Nigeria | 226.408 (161.269, 308.432) | 0.268 (0.194, 0.365) |
| Chad | 13.818 (9.457, 19.563) | 0.258 (0.181, 0.369) |
| Mali | 20.858 (14.409, 29.570) | 0.254 (0.180, 0.358) |
| Sao Tome and Principe | 0.250 (0.176, 0.349) | 0.246 (0.176, 0.345) |
| Guinea | 13.092 (9.038, 18.465) | 0.245 (0.174, 0.345) |
| Côte d’Ivoire | 24.877 (17.111, 35.193) | 0.241 (0.168, 0.338) |
| Liberia | 4.750 (3.230, 6.645) | 0.240 (0.165, 0.333) |
| Egypt | 124.044 (85.141, 170.005) | 0.239 (0.167, 0.340) |
| Mauritania | 4.781 (3.267, 6.675) | 0.238 (0.164, 0.335) |
| Sierra Leone | 8.329 (5.661, 11.672) | 0.238 (0.167, 0.336) |
| Niger | 17.381 (11.901, 25.119) | 0.236 (0.167, 0.340) |
| Senegal | 17.057 (11.516, 24.517) | 0.235 (0.161, 0.338) |
| Cameroon | 26.589 (17.909, 37.736) | 0.229 (0.160, 0.322) |
| Gambia | 2.175 (1.475, 3.097) | 0.229 (0.158, 0.324) |
| Qatar | 2.070 (1.221, 2.954) | 0.229 (0.152, 0.321) |
| France | 385.568 (288.285, 503.276) | 0.224 (0.168, 0.293) |
| Benin | 10.666 (7.407, 14.990) | 0.223 (0.157, 0.310) |
| Spain | 269.734 (199.855, 345.775) | 0.222 (0.167, 0.285) |
| Burkina Faso | 19.274 (13.296, 26.751) | 0.219 (0.152, 0.301) |
| Guinea-Bissau | 1.529 (1.057, 2.146) | 0.217 (0.153, 0.300) |
| Saudi Arabia | 31.505 (20.999, 43.830) | 0.212 (0.147, 0.295) |
| Bahrain | 1.714 (1.094, 2.410) | 0.207 (0.146, 0.287) |
| Ghana | 32.470 (22.329, 46.376) | 0.207 (0.144, 0.294) |
| Sudan | 34.419 (23.762, 48.272) | 0.206 (0.143, 0.293) |
| Togo | 7.422 (5.011, 10.506) | 0.206 (0.146, 0.285) |
| Cabo Verde | 0.880 (0.614, 1.227) | 0.205 (0.143, 0.293) |
| Algeria | 59.931 (41.823, 82.370) | 0.204 (0.144, 0.282) |
| Lebanon | 10.350 (7.437, 13.898) | 0.199 (0.143, 0.266) |
| Libya | 9.118 (6.429, 12.160) | 0.197 (0.140, 0.268) |
| Yemen | 23.307 (16.501, 32.337) | 0.197 (0.143, 0.272) |
| Oman | 3.223 (2.074, 4.455) | 0.191 (0.133, 0.264) |
| Iraq | 38.450 (27.059, 52.645) | 0.189 (0.135, 0.265) |
| Tunisia | 22.210 (15.229, 30.649) | 0.188 (0.131, 0.258) |
| Kuwait | 4.447 (3.024, 6.119) | 0.187 (0.129, 0.271) |
| Morocco | 51.781 (35.464, 70.180) | 0.187 (0.133, 0.258) |
| Afghanistan | 19.487 (13.318, 26.698) | 0.179 (0.126, 0.244) |
| Syrian Arab Republic | 18.380 (12.348, 24.978) | 0.178 (0.122, 0.242) |
| Germany | 429.267 (310.696, 575.891) | 0.172 (0.125, 0.227) |
| Jordan | 9.759 (6.506, 13.461) | 0.169 (0.116, 0.235) |
| Palestine | 3.256 (2.123, 4.464) | 0.149 (0.100, 0.204) |
| Austria | 21.913 (16.614, 28.917) | 0.110 (0.084, 0.146) |
| Bermuda | 0.146 (0.106, 0.205) | 0.107 (0.079, 0.147) |
| Monaco | 0.079 (0.056, 0.107) | 0.076 (0.053, 0.101) |
| Bahamas | 0.248 (0.155, 0.438) | 0.070 (0.045, 0.133) |
| Luxembourg | 0.751 (0.540, 1.021) | 0.070 (0.050, 0.096) |
| Sweden | 16.636 (9.953, 35.897) | 0.069 (0.043, 0.143) |
| Haiti | 3.781 (2.311, 5.809) | 0.061 (0.038, 0.101) |
| Saint Vincent and the Grenadines | 0.073 (0.046, 0.125) | 0.058 (0.037, 0.098) |
| Cuba | 9.957 (6.685, 15.868) | 0.053 (0.035, 0.084) |
| Guyana | 0.303 (0.193, 0.469) | 0.053 (0.034, 0.081) |
| Suriname | 0.282 (0.188, 0.425) | 0.049 (0.033, 0.073) |
| Switzerland | 9.165 (6.388, 12.838) | 0.045 (0.032, 0.063) |
| San Marino | 0.032 (0.023, 0.050) | 0.044 (0.032, 0.065) |
| Belize | 0.115 (0.073, 0.173) | 0.041 (0.027, 0.061) |
| Dominica | 0.036 (0.024, 0.054) | 0.041 (0.027, 0.062) |
| Grenada | 0.043 (0.027, 0.067) | 0.041 (0.027, 0.062) |
| Saint Lucia | 0.085 (0.055, 0.127) | 0.041 (0.027, 0.061) |
| Belgium | 10.246 (7.346, 14.300) | 0.040 (0.028, 0.055) |
| United States Virgin Islands | 0.066 (0.041, 0.103) | 0.040 (0.025, 0.059) |
| Saint Kitts and Nevis | 0.024 (0.015, 0.037) | 0.039 (0.025, 0.057) |
| Barbados | 0.168 (0.109, 0.254) | 0.037 (0.023, 0.054) |
| Dominican Republic | 3.475 (2.181, 5.083) | 0.037 (0.024, 0.053) |
| Antigua and Barbuda | 0.035 (0.021, 0.053) | 0.036 (0.022, 0.053) |
| Trinidad and Tobago | 0.630 (0.387, 0.939) | 0.036 (0.022, 0.053) |
| Jamaica | 1.092 (0.705, 1.595) | 0.035 (0.022, 0.052) |
| Puerto Rico | 2.314 (1.505, 3.432) | 0.035 (0.022, 0.052) |
| Finland | 4.342 (3.049, 5.879) | 0.033 (0.024, 0.046) |
| Netherlands | 9.904 (6.737, 13.857) | 0.028 (0.019, 0.040) |
| Andorra | 0.038 (0.026, 0.053) | 0.027 (0.018, 0.037) |
| Cyprus | 0.459 (0.308, 0.675) | 0.027 (0.019, 0.038) |
| Denmark | 0.809 (0.401, 1.333) | 0.009 (0.004, 0.015) |
| Greece | 1.704 (0.914, 2.904) | 0.009 (0.004, 0.015) |
| Israel | 0.974 (0.464, 1.601) | 0.009 (0.004, 0.015) |
| Iceland | 0.037 (0.017, 0.062) | 0.008 (0.004, 0.013) |
| Ireland | 0.535 (0.254, 0.893) | 0.008 (0.004, 0.014) |
| Malta | 0.060 (0.029, 0.098) | 0.008 (0.004, 0.014) |
