# Supplementary material for: Current status, trends, and predictions in the burden of silicosis in 204 countries and territories from 1990 to 2019
Source: Front Public Health. 2023 Jul 13;11:1216924. doi: 10.3389/fpubh.2023.1216924 (PMC10372342; doi:10.3389/fpubh.2023.1216924)
Supplement: Supplementary file 3 [file Table_3.DOCX]

**Table S3.** Numbers and ASRs of incidence for silicosis in nations in 2019 (per 100,000 Population)

| **location** | **Location** | **ASR s per 100 000 (95% UI)** |
| --- | --- | --- |
| China | 120774.592 (98044.363, 145938.948) | 5.918 (4.866, 7.073) |
| Democratic People's Republic of Korea | 1071.471 (800.419, 1463.574) | 3.150 (2.384, 4.313) |
| Chile | 358.347 (279.530, 459.486) | 1.472 (1.147, 1.889) |
| Italy | 1668.150 (1296.750, 2109.673) | 1.194 (0.961, 1.495) |
| Mexico | 1022.742 (815.780, 1294.334) | 0.810 (0.642, 1.018) |
| Palau | 0.157 (0.117, 0.204) | 0.657 (0.506, 0.829) |
| Japan | 2381.535 (1882.462, 2954.903) | 0.655 (0.521, 0.817) |
| Brazil | 1504.357 (1147.344, 1981.254) | 0.613 (0.470, 0.807) |
| Hungary | 84.768 (65.892, 109.212) | 0.560 (0.434, 0.718) |
| Albania | 21.111 (15.044, 32.058) | 0.559 (0.413, 0.813) |
| Slovenia | 15.557 (11.331, 20.834) | 0.520 (0.387, 0.683) |
| Slovakia | 36.719 (26.818, 49.209) | 0.453 (0.337, 0.598) |
| South Africa | 216.969 (170.351, 268.176) | 0.450 (0.360, 0.558) |
| Taiwan (Province of China) | 178.624 (145.601, 220.082) | 0.447 (0.365, 0.547) |
| Kiribati | 0.365 (0.262, 0.517) | 0.439 (0.327, 0.627) |
| Paraguay | 28.139 (21.998, 35.250) | 0.429 (0.337, 0.536) |
| Romania | 120.755 (88.434, 159.430) | 0.425 (0.317, 0.561) |
| Lesotho | 5.799 (4.319, 9.579) | 0.416 (0.318, 0.663) |
| Bulgaria | 43.347 (32.867, 55.502) | 0.407 (0.310, 0.521) |
| Czechia | 64.649 (49.269, 82.494) | 0.403 (0.309, 0.511) |
| Eswatini | 2.491 (1.863, 3.719) | 0.394 (0.298, 0.580) |
| Croatia | 23.423 (16.919, 31.036) | 0.377 (0.278, 0.489) |
| Botswana | 5.564 (4.001, 7.514) | 0.353 (0.259, 0.462) |
| India | 4270.659 (3444.658, 5336.688) | 0.346 (0.276, 0.437) |
| Papua New Guinea | 20.673 (15.292, 27.237) | 0.341 (0.257, 0.444) |
| Norway | 26.064 (19.890, 33.814) | 0.339 (0.263, 0.430) |
| Latvia | 8.841 (6.581, 11.398) | 0.337 (0.255, 0.435) |
| Guam | 0.616 (0.432, 0.832) | 0.318 (0.226, 0.425) |
| Serbia | 38.935 (27.834, 52.281) | 0.318 (0.234, 0.427) |
| Fiji | 2.753 (1.937, 3.665) | 0.316 (0.226, 0.416) |
| American Samoa | 0.166 (0.116, 0.224) | 0.315 (0.228, 0.423) |
| Northern Mariana Islands | 0.195 (0.131, 0.274) | 0.315 (0.227, 0.419) |
| Vanuatu | 0.616 (0.455, 0.823) | 0.314 (0.233, 0.423) |
| Bosnia and Herzegovina | 15.572 (11.031, 20.842) | 0.312 (0.226, 0.413) |
| Marshall Islands | 0.135 (0.099, 0.178) | 0.311 (0.231, 0.409) |
| North Macedonia | 9.344 (6.836, 12.179) | 0.311 (0.232, 0.402) |
| Namibia | 4.731 (3.516, 6.092) | 0.310 (0.233, 0.396) |
| Tokelau | 0.004 (0.003, 0.006) | 0.309 (0.226, 0.410) |
| Samoa | 0.502 (0.364, 0.665) | 0.308 (0.226, 0.407) |
| Ukraine | 190.516 (139.804, 252.548) | 0.308 (0.228, 0.402) |
| Montenegro | 2.591 (1.841, 3.498) | 0.303 (0.219, 0.406) |
| Solomon Islands | 1.203 (0.891, 1.584) | 0.303 (0.231, 0.397) |
| Estonia | 5.364 (3.790, 7.211) | 0.302 (0.218, 0.404) |
| Cook Islands | 0.072 (0.050, 0.097) | 0.300 (0.217, 0.402) |
| Maldives | 1.390 (1.006, 1.870) | 0.300 (0.214, 0.395) |
| Argentina | 161.691 (121.773, 215.491) | 0.299 (0.228, 0.397) |
| Republic of Moldova | 15.109 (10.675, 20.532) | 0.298 (0.216, 0.402) |
| Micronesia (Federated States of) | 0.254 (0.179, 0.346) | 0.296 (0.216, 0.391) |
| Uruguay | 15.312 (10.917, 21.131) | 0.296 (0.212, 0.409) |
| Niue | 0.006 (0.004, 0.009) | 0.295 (0.210, 0.395) |
| Tonga | 0.243 (0.183, 0.320) | 0.295 (0.221, 0.386) |
| Tuvalu | 0.032 (0.023, 0.043) | 0.294 (0.215, 0.399) |
| Belarus | 38.382 (27.693, 50.992) | 0.291 (0.215, 0.377) |
| Nauru | 0.018 (0.013, 0.024) | 0.280 (0.207, 0.370) |
| Lithuania | 11.306 (8.316, 15.084) | 0.278 (0.209, 0.365) |
| Russian Federation | 538.669 (393.813, 718.312) | 0.275 (0.202, 0.361) |
| Malaysia | 80.807 (57.711, 107.093) | 0.266 (0.190, 0.352) |
| Seychelles | 0.325 (0.226, 0.444) | 0.259 (0.184, 0.348) |
| Timor-Leste | 2.297 (1.658, 3.048) | 0.259 (0.187, 0.343) |
| Mauritius | 4.458 (3.049, 6.076) | 0.253 (0.181, 0.335) |
| Thailand | 257.558 (181.570, 346.318) | 0.251 (0.182, 0.332) |
| Zimbabwe | 19.286 (15.041, 24.586) | 0.249 (0.191, 0.317) |
| Bangladesh | 330.512 (248.744, 424.889) | 0.247 (0.187, 0.317) |
| Indonesia | 662.851 (481.462, 886.927) | 0.246 (0.180, 0.327) |
| Lao People's Democratic Republic | 13.689 (9.868, 18.361) | 0.245 (0.178, 0.328) |
| Sri Lanka | 63.362 (45.021, 85.742) | 0.242 (0.174, 0.325) |
| Nepal | 56.499 (44.130, 71.709) | 0.240 (0.188, 0.304) |
| Singapore | 18.633 (13.310, 25.974) | 0.240 (0.173, 0.335) |
| Viet Nam | 253.902 (181.472, 341.744) | 0.230 (0.167, 0.307) |
| New Zealand | 18.095 (12.967, 24.773) | 0.224 (0.162, 0.309) |
| Republic of Korea | 201.400 (146.930, 268.991) | 0.224 (0.163, 0.299) |
| Myanmar | 118.795 (86.208, 157.501) | 0.220 (0.162, 0.290) |
| Cambodia | 31.270 (22.845, 41.429) | 0.219 (0.161, 0.291) |
| Bhutan | 1.261 (0.984, 1.613) | 0.218 (0.170, 0.280) |
| Brunei Darussalam | 0.529 (0.372, 0.746) | 0.215 (0.155, 0.301) |
| Philippines | 198.915 (148.679, 272.027) | 0.210 (0.157, 0.281) |
| Burundi | 8.096 (5.772, 10.844) | 0.175 (0.125, 0.233) |
| Madagascar | 18.983 (13.182, 26.096) | 0.175 (0.126, 0.237) |
| Somalia | 10.931 (7.633, 15.727) | 0.164 (0.116, 0.235) |
| Djibouti | 0.963 (0.698, 1.290) | 0.160 (0.117, 0.213) |
| Pakistan | 192.361 (147.953, 247.756) | 0.160 (0.124, 0.205) |
| Central African Republic | 3.342 (2.480, 4.451) | 0.156 (0.121, 0.203) |
| Democratic Republic of the Congo | 54.609 (40.152, 71.040) | 0.156 (0.117, 0.202) |
| Kenya | 34.952 (27.192, 45.566) | 0.154 (0.121, 0.199) |
| Mozambique | 17.123 (12.074, 24.505) | 0.154 (0.110, 0.217) |
| Rwanda | 9.386 (6.504, 14.221) | 0.152 (0.107, 0.229) |
| Malawi | 10.657 (7.620, 14.109) | 0.147 (0.106, 0.194) |
| Angola | 14.986 (11.226, 19.768) | 0.135 (0.103, 0.176) |
| Zambia | 8.811 (6.497, 11.701) | 0.135 (0.100, 0.179) |
| South Sudan | 5.062 (3.869, 6.542) | 0.134 (0.104, 0.172) |
| Comoros | 0.634 (0.469, 0.824) | 0.132 (0.098, 0.171) |
| Congo | 3.352 (2.551, 4.425) | 0.132 (0.101, 0.172) |
| Uganda | 18.267 (13.419, 23.969) | 0.132 (0.098, 0.173) |
| Australia | 57.778 (39.435, 99.078) | 0.128 (0.088, 0.217) |
| Ethiopia | 51.551 (40.060, 66.199) | 0.125 (0.096, 0.161) |
| Armenia | 5.420 (3.793, 9.376) | 0.124 (0.089, 0.209) |
| United Republic of Tanzania | 29.901 (22.881, 39.110) | 0.123 (0.095, 0.161) |
| United Kingdom | 131.513 (97.610, 174.912) | 0.122 (0.092, 0.160) |
| Eritrea | 3.288 (2.412, 4.399) | 0.121 (0.090, 0.159) |
| Colombia | 62.191 (43.431, 100.988) | 0.119 (0.083, 0.193) |
| Equatorial Guinea | 0.547 (0.421, 0.717) | 0.119 (0.091, 0.157) |
| Gabon | 1.257 (0.943, 1.700) | 0.119 (0.091, 0.158) |
| Canada | 69.874 (49.795, 116.952) | 0.104 (0.075, 0.168) |
| Bolivia (Plurinational State of) | 9.323 (6.417, 16.044) | 0.098 (0.067, 0.168) |
| Honduras | 4.841 (3.480, 6.578) | 0.076 (0.055, 0.104) |
| Poland | 43.284 (35.300, 54.256) | 0.075 (0.063, 0.091) |
| Ecuador | 11.549 (8.348, 16.037) | 0.072 (0.052, 0.101) |
| Peru | 23.012 (16.166, 33.361) | 0.070 (0.049, 0.102) |
| Mongolia | 1.895 (1.295, 2.627) | 0.068 (0.049, 0.093) |
| Tajikistan | 4.245 (2.911, 6.019) | 0.068 (0.049, 0.095) |
| United States of America | 335.805 (256.087, 445.166) | 0.064 (0.050, 0.084) |
| Azerbaijan | 7.178 (4.775, 10.354) | 0.062 (0.044, 0.086) |
| Uzbekistan | 16.698 (11.038, 23.135) | 0.062 (0.044, 0.084) |
| Costa Rica | 3.211 (2.345, 4.423) | 0.061 (0.045, 0.085) |
| Georgia | 3.478 (2.371, 4.913) | 0.061 (0.042, 0.085) |
| Turkmenistan | 2.776 (1.916, 4.068) | 0.061 (0.043, 0.089) |
| Kyrgyzstan | 3.196 (2.150, 4.592) | 0.060 (0.042, 0.085) |
| Kazakhstan | 11.258 (7.690, 16.240) | 0.058 (0.041, 0.083) |
| Nicaragua | 2.635 (1.894, 3.632) | 0.057 (0.040, 0.078) |
| Panama | 2.341 (1.657, 3.311) | 0.057 (0.040, 0.080) |
| Venezuela (Bolivarian Republic of) | 16.865 (11.879, 23.730) | 0.056 (0.040, 0.079) |
| Guatemala | 6.261 (4.390, 8.469) | 0.055 (0.038, 0.075) |
| El Salvador | 2.996 (2.143, 4.185) | 0.051 (0.036, 0.072) |
| Greenland | 0.032 (0.022, 0.047) | 0.046 (0.034, 0.064) |
| Portugal | 10.079 (7.792, 12.852) | 0.046 (0.036, 0.059) |
| Iran (Islamic Republic of) | 24.131 (17.879, 33.062) | 0.035 (0.027, 0.049) |
| France | 45.698 (34.059, 60.177) | 0.030 (0.023, 0.040) |
| Spain | 30.804 (22.962, 39.797) | 0.029 (0.022, 0.037) |
| Turkey | 22.544 (15.100, 39.275) | 0.026 (0.017, 0.045) |
| United Arab Emirates | 0.882 (0.540, 1.344) | 0.026 (0.018, 0.035) |
| Germany | 55.027 (39.433, 74.528) | 0.023 (0.017, 0.031) |
| Nigeria | 19.132 (14.077, 25.925) | 0.021 (0.015, 0.028) |
| Bahrain | 0.173 (0.112, 0.252) | 0.020 (0.014, 0.027) |
| Chad | 1.175 (0.830, 1.652) | 0.020 (0.014, 0.029) |
| Côte d’Ivoire | 2.162 (1.513, 3.032) | 0.019 (0.013, 0.026) |
| Egypt | 11.265 (7.865, 16.372) | 0.019 (0.013, 0.027) |
| Guinea | 1.078 (0.770, 1.545) | 0.019 (0.013, 0.027) |
| Liberia | 0.407 (0.286, 0.553) | 0.019 (0.013, 0.026) |
| Mali | 1.728 (1.224, 2.418) | 0.019 (0.013, 0.027) |
| Qatar | 0.199 (0.123, 0.291) | 0.019 (0.012, 0.026) |
| Sao Tome and Principe | 0.021 (0.015, 0.029) | 0.019 (0.013, 0.026) |
| Sierra Leone | 0.698 (0.497, 0.983) | 0.019 (0.013, 0.027) |
| Benin | 0.903 (0.652, 1.243) | 0.018 (0.012, 0.025) |
| Cameroon | 2.249 (1.589, 3.122) | 0.018 (0.013, 0.025) |
| Gambia | 0.181 (0.128, 0.251) | 0.018 (0.013, 0.026) |
| Mauritania | 0.375 (0.265, 0.513) | 0.018 (0.012, 0.025) |
| Niger | 1.481 (1.051, 2.126) | 0.018 (0.013, 0.026) |
| Saudi Arabia | 2.942 (2.015, 4.140) | 0.018 (0.012, 0.026) |
| Senegal | 1.401 (0.973, 2.015) | 0.018 (0.012, 0.026) |
| Sudan | 3.131 (2.248, 4.378) | 0.018 (0.013, 0.025) |
| Algeria | 5.308 (3.689, 7.469) | 0.017 (0.012, 0.024) |
| Burkina Faso | 1.638 (1.147, 2.256) | 0.017 (0.012, 0.024) |
| Guinea-Bissau | 0.133 (0.094, 0.184) | 0.017 (0.012, 0.024) |
| Iraq | 3.539 (2.551, 4.975) | 0.017 (0.012, 0.023) |
| Lebanon | 0.869 (0.625, 1.171) | 0.017 (0.012, 0.023) |
| Libya | 0.796 (0.576, 1.086) | 0.017 (0.012, 0.024) |
| Oman | 0.300 (0.201, 0.415) | 0.017 (0.011, 0.023) |
| Yemen | 2.191 (1.599, 2.988) | 0.017 (0.013, 0.024) |
| Cabo Verde | 0.070 (0.050, 0.097) | 0.016 (0.011, 0.022) |
| Ghana | 2.709 (1.906, 3.825) | 0.016 (0.011, 0.023) |
| Morocco | 4.697 (3.282, 6.530) | 0.016 (0.011, 0.022) |
| Togo | 0.642 (0.459, 0.899) | 0.016 (0.012, 0.022) |
| Tunisia | 1.979 (1.376, 2.782) | 0.016 (0.011, 0.023) |
| Afghanistan | 1.761 (1.268, 2.335) | 0.015 (0.011, 0.021) |
| Kuwait | 0.376 (0.264, 0.535) | 0.015 (0.010, 0.023) |
| Jordan | 0.859 (0.591, 1.201) | 0.014 (0.010, 0.020) |
| Syrian Arab Republic | 1.623 (1.094, 2.281) | 0.014 (0.010, 0.020) |
| Bermuda | 0.018 (0.013, 0.024) | 0.013 (0.010, 0.017) |
| Palestine | 0.298 (0.202, 0.409) | 0.013 (0.009, 0.018) |
| Austria | 2.301 (1.692, 3.064) | 0.012 (0.009, 0.016) |
| Sweden | 2.313 (1.258, 5.026) | 0.009 (0.005, 0.020) |
| Luxembourg | 0.083 (0.059, 0.116) | 0.008 (0.006, 0.011) |
| Bahamas | 0.024 (0.015, 0.049) | 0.007 (0.004, 0.015) |
| Monaco | 0.007 (0.005, 0.009) | 0.007 (0.005, 0.009) |
| Haiti | 0.351 (0.225, 0.603) | 0.006 (0.004, 0.011) |
| Switzerland | 1.137 (0.792, 1.578) | 0.006 (0.004, 0.008) |
| Belgium | 1.178 (0.839, 1.629) | 0.005 (0.003, 0.006) |
| Cuba | 0.900 (0.562, 1.604) | 0.005 (0.003, 0.008) |
| Guyana | 0.026 (0.016, 0.042) | 0.005 (0.003, 0.008) |
| Saint Vincent and the Grenadines | 0.007 (0.004, 0.013) | 0.005 (0.003, 0.010) |
| San Marino | 0.004 (0.003, 0.007) | 0.005 (0.004, 0.008) |
| Suriname | 0.023 (0.015, 0.037) | 0.004 (0.003, 0.007) |
| Andorra | 0.004 (0.003, 0.006) | 0.003 (0.002, 0.004) |
| Antigua and Barbuda | 0.002 (0.002, 0.004) | 0.003 (0.002, 0.004) |
| Barbados | 0.012 (0.008, 0.019) | 0.003 (0.002, 0.004) |
| Belize | 0.009 (0.006, 0.013) | 0.003 (0.002, 0.005) |
| Cyprus | 0.062 (0.043, 0.090) | 0.003 (0.002, 0.005) |
| Dominica | 0.003 (0.002, 0.004) | 0.003 (0.002, 0.005) |
| Dominican Republic | 0.251 (0.170, 0.373) | 0.003 (0.002, 0.004) |
| Finland | 0.450 (0.310, 0.632) | 0.003 (0.002, 0.005) |
| Grenada | 0.004 (0.002, 0.005) | 0.003 (0.002, 0.005) |
| Jamaica | 0.082 (0.054, 0.122) | 0.003 (0.002, 0.004) |
| Netherlands | 1.242 (0.855, 1.729) | 0.003 (0.002, 0.005) |
| Saint Kitts and Nevis | 0.002 (0.001, 0.003) | 0.003 (0.002, 0.004) |
| Saint Lucia | 0.006 (0.004, 0.010) | 0.003 (0.002, 0.005) |
| Trinidad and Tobago | 0.044 (0.029, 0.067) | 0.003 (0.002, 0.004) |
| United States Virgin Islands | 0.005 (0.003, 0.008) | 0.003 (0.002, 0.004) |
| Puerto Rico | 0.168 (0.107, 0.256) | 0.002 (0.002, 0.004) |
| Denmark | 0.038 (0.021, 0.072) | 0.000 (0.000, 0.001) |
| Greece | 0.083 (0.047, 0.155) | 0.000 (0.000, 0.001) |
| Iceland | 0.002 (0.001, 0.003) | 0.000 (0.000, 0.001) |
| Ireland | 0.024 (0.013, 0.041) | 0.000 (0.000, 0.001) |
| Israel | 0.049 (0.027, 0.085) | 0.000 (0.000, 0.001) |
| Malta | 0.003 (0.001, 0.005) | 0.000 (0.000, 0.001) |
