# Supplementary material for: Current status, trends, and predictions in the burden of silicosis in 204 countries and territories from 1990 to 2019
Source: Front Public Health. 2023 Jul 13;11:1216924. doi: 10.3389/fpubh.2023.1216924 (PMC10372342; doi:10.3389/fpubh.2023.1216924)
Supplement: Supplementary file 4 [file Table_4.DOCX]

**Table S4.** Numbers and ASRs of mortality for silicosis in nations in 2019 (per 100,000 Population)

| **location** | **Location** | **ASR s per 100 000 (95% UI)** |
| --- | --- | --- |
| Palau | 0.078 (0.046, 0.133) | 0.604 (0.360, 1.013) |
| Democratic People's Republic of Korea | 192.632 (85.868, 341.649) | 0.599 (0.271, 1.049) |
| Chile | 123.009 (100.823, 153.339) | 0.511 (0.420, 0.635) |
| China | 7746.160 (5938.688, 10860.088) | 0.404 (0.312, 0.564) |
| Portugal | 96.089 (79.682, 116.083) | 0.366 (0.305, 0.439) |
| Taiwan (Province of China) | 121.422 (84.565, 162.702) | 0.308 (0.213, 0.415) |
| Nepal | 53.525 (11.559, 98.069) | 0.269 (0.059, 0.478) |
| Lesotho | 3.214 (1.414, 5.628) | 0.250 (0.116, 0.441) |
| Somalia | 15.530 (0.986, 44.735) | 0.249 (0.017, 0.705) |
| Paraguay | 13.142 (8.355, 18.452) | 0.220 (0.141, 0.308) |
| Kiribati | 0.076 (0.041, 0.122) | 0.211 (0.111, 0.350) |
| France | 357.540 (261.677, 507.503) | 0.208 (0.153, 0.293) |
| Italy | 388.873 (331.634, 437.492) | 0.203 (0.173, 0.229) |
| Spain | 210.530 (162.550, 280.166) | 0.177 (0.138, 0.234) |
| Central African Republic | 3.474 (0.391, 8.657) | 0.170 (0.024, 0.409) |
| Eswatini | 0.846 (0.362, 1.477) | 0.147 (0.069, 0.253) |
| Germany | 342.293 (257.647, 488.640) | 0.140 (0.106, 0.205) |
| Pakistan | 129.628 (47.311, 204.804) | 0.138 (0.053, 0.214) |
| India | 1431.129 (620.420, 2202.911) | 0.137 (0.061, 0.209) |
| Burundi | 5.018 (0.537, 13.000) | 0.123 (0.014, 0.314) |
| Brazil | 277.553 (252.302, 314.413) | 0.117 (0.107, 0.133) |
| Namibia | 1.573 (0.820, 2.620) | 0.117 (0.062, 0.194) |
| Albania | 5.089 (2.847, 9.693) | 0.115 (0.065, 0.218) |
| Honduras | 6.533 (2.026, 11.360) | 0.113 (0.035, 0.196) |
| Botswana | 1.478 (0.774, 2.601) | 0.112 (0.060, 0.200) |
| Madagascar | 10.006 (1.696, 22.582) | 0.106 (0.018, 0.240) |
| Hungary | 20.858 (15.881, 26.272) | 0.102 (0.077, 0.128) |
| Democratic Republic of the Congo | 30.511 (4.639, 78.044) | 0.098 (0.016, 0.248) |
| Mozambique | 9.879 (1.586, 22.036) | 0.096 (0.017, 0.211) |
| South Africa | 39.480 (27.563, 50.715) | 0.093 (0.064, 0.118) |
| Monaco | 0.099 (0.056, 0.173) | 0.092 (0.052, 0.159) |
| Bolivia (Plurinational State of) | 7.660 (3.841, 12.299) | 0.091 (0.047, 0.144) |
| Solomon Islands | 0.154 (0.048, 0.313) | 0.086 (0.022, 0.178) |
| Japan | 412.729 (292.565, 769.038) | 0.085 (0.061, 0.159) |
| San Marino | 0.063 (0.037, 0.099) | 0.085 (0.050, 0.132) |
| Bangladesh | 103.537 (22.859, 184.309) | 0.084 (0.019, 0.150) |
| Bhutan | 0.410 (0.083, 0.790) | 0.083 (0.017, 0.157) |
| Eritrea | 1.931 (0.332, 4.661) | 0.078 (0.016, 0.183) |
| Malawi | 5.210 (0.917, 11.917) | 0.078 (0.014, 0.177) |
| Austria | 15.139 (12.079, 19.991) | 0.077 (0.062, 0.101) |
| Mexico | 84.432 (67.251, 105.078) | 0.075 (0.060, 0.094) |
| Papua New Guinea | 2.019 (0.424, 4.761) | 0.074 (0.014, 0.168) |
| Rwanda | 3.944 (1.182, 7.732) | 0.074 (0.023, 0.141) |
| Uganda | 9.150 (1.789, 18.601) | 0.072 (0.015, 0.144) |
| Colombia | 36.652 (26.182, 49.705) | 0.069 (0.050, 0.094) |
| Luxembourg | 0.727 (0.535, 0.964) | 0.069 (0.051, 0.092) |
| Romania | 24.626 (18.621, 31.853) | 0.065 (0.049, 0.084) |
| Zimbabwe | 3.625 (2.106, 5.817) | 0.064 (0.036, 0.107) |
| Zambia | 3.747 (0.729, 7.582) | 0.063 (0.013, 0.125) |
| Comoros | 0.265 (0.055, 0.594) | 0.059 (0.012, 0.132) |
| Ethiopia | 21.497 (3.496, 49.620) | 0.059 (0.010, 0.136) |
| Haiti | 3.373 (0.625, 8.314) | 0.058 (0.010, 0.146) |
| Vanuatu | 0.066 (0.015, 0.145) | 0.058 (0.013, 0.124) |
| South Sudan | 1.806 (0.404, 4.309) | 0.056 (0.013, 0.131) |
| Slovakia | 5.240 (3.727, 7.196) | 0.055 (0.039, 0.075) |
| Bulgaria | 8.393 (5.505, 13.341) | 0.054 (0.035, 0.087) |
| Belgium | 13.348 (9.302, 18.621) | 0.051 (0.036, 0.070) |
| Congo | 1.085 (0.257, 2.104) | 0.050 (0.014, 0.093) |
| Kenya | 9.822 (2.745, 17.950) | 0.050 (0.015, 0.091) |
| United Republic of Tanzania | 11.118 (2.985, 22.611) | 0.050 (0.014, 0.100) |
| Djibouti | 0.241 (0.068, 0.540) | 0.048 (0.015, 0.106) |
| Angola | 4.379 (1.213, 9.707) | 0.047 (0.015, 0.098) |
| Switzerland | 9.492 (7.241, 12.901) | 0.046 (0.035, 0.062) |
| Canada | 31.173 (19.727, 78.742) | 0.044 (0.029, 0.107) |
| Marshall Islands | 0.008 (0.003, 0.016) | 0.043 (0.015, 0.090) |
| Bermuda | 0.054 (0.041, 0.069) | 0.040 (0.031, 0.051) |
| Micronesia (Federated States of) | 0.017 (0.008, 0.035) | 0.039 (0.017, 0.078) |
| Andorra | 0.057 (0.016, 0.214) | 0.038 (0.010, 0.141) |
| Czechia | 8.506 (5.551, 12.317) | 0.037 (0.025, 0.053) |
| Bahamas | 0.123 (0.091, 0.163) | 0.035 (0.026, 0.046) |
| Argentina | 18.131 (14.409, 22.227) | 0.034 (0.027, 0.041) |
| Nauru | 0.001 (0.000, 0.001) | 0.032 (0.016, 0.056) |
| Tuvalu | 0.002 (0.001, 0.005) | 0.032 (0.013, 0.065) |
| Finland | 4.347 (2.221, 18.703) | 0.031 (0.016, 0.133) |
| Gabon | 0.280 (0.078, 0.638) | 0.031 (0.009, 0.067) |
| Samoa | 0.036 (0.020, 0.066) | 0.031 (0.017, 0.057) |
| Afghanistan | 3.761 (0.738, 7.560) | 0.030 (0.006, 0.058) |
| Australia | 13.352 (7.649, 44.690) | 0.029 (0.017, 0.096) |
| Equatorial Guinea | 0.113 (0.034, 0.287) | 0.029 (0.009, 0.074) |
| Netherlands | 10.848 (7.656, 18.585) | 0.029 (0.021, 0.049) |
| Peru | 9.226 (4.573, 18.745) | 0.029 (0.014, 0.059) |
| Turkey | 24.049 (15.514, 45.187) | 0.027 (0.017, 0.052) |
| Armenia | 1.077 (0.787, 1.448) | 0.026 (0.019, 0.034) |
| Cyprus | 0.457 (0.292, 0.814) | 0.026 (0.017, 0.045) |
| Yemen | 3.309 (0.829, 6.181) | 0.026 (0.007, 0.049) |
| Ecuador | 3.583 (2.250, 5.858) | 0.025 (0.016, 0.040) |
| Republic of Korea | 22.764 (8.974, 109.330) | 0.025 (0.010, 0.122) |
| Tonga | 0.018 (0.008, 0.035) | 0.024 (0.011, 0.047) |
| Uruguay | 1.116 (0.907, 1.329) | 0.024 (0.019, 0.028) |
| Guinea-Bissau | 0.225 (0.077, 0.451) | 0.023 (0.008, 0.045) |
| Iran (Islamic Republic of) | 15.382 (6.172, 19.985) | 0.023 (0.009, 0.030) |
| Mali | 2.425 (1.028, 4.176) | 0.023 (0.009, 0.039) |
| Sao Tome and Principe | 0.028 (0.013, 0.049) | 0.023 (0.011, 0.039) |
| Nicaragua | 0.827 (0.610, 1.134) | 0.022 (0.016, 0.030) |
| Sudan | 3.917 (0.781, 8.029) | 0.022 (0.004, 0.045) |
| Montenegro | 0.203 (0.106, 0.353) | 0.021 (0.011, 0.036) |
| Chad | 1.281 (0.465, 2.353) | 0.020 (0.006, 0.035) |
| Egypt | 12.230 (2.554, 25.396) | 0.020 (0.004, 0.040) |
| Guinea | 1.266 (0.510, 2.197) | 0.020 (0.008, 0.033) |
| Niger | 1.773 (0.477, 3.499) | 0.020 (0.005, 0.038) |
| Saint Vincent and the Grenadines | 0.025 (0.018, 0.034) | 0.020 (0.014, 0.027) |
| Suriname | 0.112 (0.064, 0.176) | 0.020 (0.011, 0.031) |
| Costa Rica | 0.944 (0.649, 1.332) | 0.018 (0.013, 0.025) |
| Guyana | 0.108 (0.077, 0.147) | 0.018 (0.013, 0.025) |
| Tokelau | 0.000 (0.000, 0.000) | 0.018 (0.009, 0.033) |
| Ukraine | 13.524 (9.062, 19.744) | 0.017 (0.011, 0.025) |
| United States of America | 98.454 (82.945, 140.872) | 0.017 (0.015, 0.024) |
| Iraq | 3.325 (1.701, 5.618) | 0.016 (0.009, 0.027) |
| Israel | 1.703 (1.367, 2.164) | 0.016 (0.013, 0.020) |
| Niue | 0.000 (0.000, 0.001) | 0.016 (0.008, 0.027) |
| Sierra Leone | 0.689 (0.242, 1.307) | 0.016 (0.005, 0.030) |
| United Arab Emirates | 0.892 (0.106, 2.935) | 0.016 (0.002, 0.059) |
| Gambia | 0.167 (0.057, 0.313) | 0.015 (0.005, 0.028) |
| Serbia | 2.472 (1.598, 4.386) | 0.015 (0.010, 0.026) |
| Sweden | 3.687 (2.417, 8.494) | 0.015 (0.010, 0.034) |
| Togo | 0.691 (0.280, 1.284) | 0.015 (0.006, 0.027) |
| Benin | 0.842 (0.340, 1.615) | 0.014 (0.006, 0.025) |
| Morocco | 3.906 (1.132, 6.752) | 0.014 (0.004, 0.024) |
| Poland | 10.214 (7.770, 14.391) | 0.014 (0.011, 0.020) |
| Cameroon | 1.961 (0.831, 3.864) | 0.013 (0.005, 0.023) |
| Côte d’Ivoire | 1.825 (0.797, 3.603) | 0.013 (0.006, 0.024) |
| Senegal | 1.091 (0.473, 1.936) | 0.013 (0.006, 0.022) |
| North Macedonia | 0.387 (0.269, 0.560) | 0.012 (0.009, 0.017) |
| Norway | 1.244 (0.995, 2.229) | 0.012 (0.010, 0.022) |
| Oman | 0.154 (0.049, 0.286) | 0.012 (0.004, 0.023) |
| United Kingdom | 17.235 (13.120, 34.310) | 0.012 (0.009, 0.025) |
| Algeria | 3.007 (1.119, 4.973) | 0.011 (0.004, 0.018) |
| Burkina Faso | 1.175 (0.419, 2.208) | 0.011 (0.004, 0.019) |
| Myanmar | 4.298 (1.489, 8.308) | 0.011 (0.004, 0.020) |
| Belize | 0.033 (0.023, 0.048) | 0.010 (0.007, 0.014) |
| Cuba | 1.909 (1.410, 2.468) | 0.010 (0.007, 0.013) |
| Latvia | 0.428 (0.315, 0.579) | 0.010 (0.007, 0.013) |
| Lebanon | 0.522 (0.160, 1.402) | 0.010 (0.003, 0.027) |
| Libya | 0.497 (0.162, 0.872) | 0.010 (0.004, 0.018) |
| Venezuela (Bolivarian Republic of) | 2.659 (1.790, 3.745) | 0.010 (0.006, 0.013) |
| Brunei Darussalam | 0.018 (0.013, 0.028) | 0.009 (0.006, 0.015) |
| Liberia | 0.236 (0.079, 0.500) | 0.009 (0.003, 0.019) |
| Nigeria | 8.264 (4.817, 13.872) | 0.009 (0.005, 0.014) |
| Russian Federation | 21.979 (17.110, 35.460) | 0.009 (0.007, 0.015) |
| Slovenia | 0.416 (0.170, 2.508) | 0.009 (0.004, 0.052) |
| Tunisia | 1.107 (0.363, 2.039) | 0.009 (0.003, 0.017) |
| Belarus | 1.234 (0.679, 2.443) | 0.008 (0.004, 0.015) |
| Cabo Verde | 0.045 (0.024, 0.093) | 0.008 (0.005, 0.016) |
| Cook Islands | 0.002 (0.001, 0.003) | 0.008 (0.004, 0.015) |
| Lao People's Democratic Republic | 0.290 (0.126, 0.718) | 0.008 (0.003, 0.019) |
| Bosnia and Herzegovina | 0.433 (0.172, 0.806) | 0.007 (0.003, 0.013) |
| Croatia | 0.590 (0.254, 2.703) | 0.007 (0.003, 0.030) |
| Ghana | 1.050 (0.550, 1.868) | 0.007 (0.003, 0.011) |
| Grenada | 0.007 (0.005, 0.010) | 0.007 (0.005, 0.010) |
| Mauritania | 0.169 (0.089, 0.313) | 0.007 (0.004, 0.013) |
| Mongolia | 0.103 (0.025, 0.350) | 0.007 (0.002, 0.024) |
| Saint Kitts and Nevis | 0.004 (0.003, 0.005) | 0.007 (0.005, 0.009) |
| Saint Lucia | 0.015 (0.011, 0.021) | 0.007 (0.005, 0.010) |
| Timor-Leste | 0.048 (0.019, 0.109) | 0.007 (0.003, 0.015) |
| United States Virgin Islands | 0.011 (0.007, 0.018) | 0.007 (0.004, 0.011) |
| Cambodia | 0.598 (0.241, 1.267) | 0.006 (0.002, 0.012) |
| Qatar | 0.020 (0.012, 0.034) | 0.006 (0.003, 0.010) |
| Azerbaijan | 0.416 (0.221, 0.778) | 0.005 (0.003, 0.011) |
| Bahrain | 0.037 (0.019, 0.105) | 0.005 (0.002, 0.018) |
| Denmark | 0.574 (0.392, 0.977) | 0.005 (0.003, 0.008) |
| Dominica | 0.005 (0.003, 0.008) | 0.005 (0.003, 0.008) |
| Dominican Republic | 0.466 (0.282, 0.811) | 0.005 (0.003, 0.009) |
| El Salvador | 0.307 (0.206, 0.446) | 0.005 (0.003, 0.008) |
| Greece | 1.124 (0.849, 1.430) | 0.005 (0.003, 0.006) |
| Indonesia | 9.236 (3.720, 21.279) | 0.005 (0.002, 0.012) |
| Maldives | 0.021 (0.010, 0.033) | 0.005 (0.003, 0.008) |
| Saudi Arabia | 0.607 (0.330, 1.016) | 0.005 (0.002, 0.007) |
| Singapore | 0.367 (0.242, 0.478) | 0.005 (0.003, 0.006) |
| Thailand | 4.914 (2.982, 7.729) | 0.005 (0.003, 0.007) |
| Viet Nam | 4.281 (1.623, 8.236) | 0.005 (0.002, 0.010) |
| Guatemala | 0.379 (0.146, 0.526) | 0.004 (0.001, 0.005) |
| Ireland | 0.276 (0.148, 0.857) | 0.004 (0.002, 0.011) |
| Philippines | 2.494 (1.552, 3.730) | 0.004 (0.002, 0.006) |
| Syrian Arab Republic | 0.444 (0.202, 0.740) | 0.004 (0.002, 0.007) |
| Uzbekistan | 0.442 (0.288, 0.813) | 0.004 (0.002, 0.006) |
| American Samoa | 0.001 (0.001, 0.002) | 0.003 (0.002, 0.006) |
| Barbados | 0.013 (0.010, 0.018) | 0.003 (0.002, 0.004) |
| Jamaica | 0.099 (0.065, 0.143) | 0.003 (0.002, 0.005) |
| Kuwait | 0.052 (0.032, 0.076) | 0.003 (0.002, 0.004) |
| Lithuania | 0.149 (0.100, 0.211) | 0.003 (0.002, 0.004) |
| Malaysia | 0.824 (0.488, 1.360) | 0.003 (0.002, 0.005) |
| Northern Mariana Islands | 0.001 (0.001, 0.003) | 0.003 (0.002, 0.011) |
| Palestine | 0.059 (0.041, 0.087) | 0.003 (0.002, 0.005) |
| Panama | 0.130 (0.085, 0.214) | 0.003 (0.002, 0.005) |
| Seychelles | 0.003 (0.001, 0.009) | 0.003 (0.001, 0.009) |
| Antigua and Barbuda | 0.002 (0.001, 0.002) | 0.002 (0.001, 0.002) |
| Estonia | 0.057 (0.039, 0.079) | 0.002 (0.001, 0.003) |
| Fiji | 0.011 (0.006, 0.018) | 0.002 (0.001, 0.004) |
| Georgia | 0.174 (0.088, 0.289) | 0.002 (0.001, 0.004) |
| Greenland | 0.001 (0.001, 0.003) | 0.002 (0.001, 0.005) |
| Guam | 0.003 (0.002, 0.008) | 0.002 (0.001, 0.004) |
| Jordan | 0.093 (0.061, 0.141) | 0.002 (0.001, 0.003) |
| Malta | 0.023 (0.014, 0.051) | 0.002 (0.001, 0.005) |
| Trinidad and Tobago | 0.040 (0.025, 0.062) | 0.002 (0.001, 0.003) |
| Iceland | 0.007 (0.004, 0.010) | 0.001 (0.001, 0.002) |
| Kazakhstan | 0.129 (0.060, 0.409) | 0.001 (0.000, 0.003) |
| Mauritius | 0.020 (0.014, 0.028) | 0.001 (0.001, 0.002) |
| New Zealand | 0.107 (0.054, 0.227) | 0.001 (0.001, 0.003) |
| Puerto Rico | 0.095 (0.061, 0.141) | 0.001 (0.001, 0.002) |
| Republic of Moldova | 0.073 (0.051, 0.099) | 0.001 (0.001, 0.002) |
| Sri Lanka | 0.269 (0.142, 0.485) | 0.001 (0.001, 0.002) |
| Tajikistan | 0.046 (0.028, 0.079) | 0.001 (0.001, 0.002) |
| Turkmenistan | 0.023 (0.012, 0.048) | 0.001 (0.000, 0.001) |
| Kyrgyzstan | 0.010 (0.007, 0.015) | 0.000 (0.000, 0.000) |
