# Supplementary material for: Current status, trends, and predictions in the burden of silicosis in 204 countries and territories from 1990 to 2019
Source: Front Public Health. 2023 Jul 13;11:1216924. doi: 10.3389/fpubh.2023.1216924 (PMC10372342; doi:10.3389/fpubh.2023.1216924)
Supplement: Supplementary file 5 [file Table_5.DOCX]

**Table S5.** Numbers and ASRs of DALYs for silicosis in nations in 2019 (per 100,000 Population)

| **location** | **Location** | **ASR s per 100 000 (95% UI)** |
| --- | --- | --- |
| China | 519696.846 (391699.829, 677968.302) | 24.969 (18.900, 32.513) |
| Democratic People's Republic of Korea | 7782.319 (4451.937, 12294.017) | 23.475 (13.458, 36.796) |
| Palau | 2.464 (1.442, 3.965) | 13.114 (7.961, 20.627) |
| Chile | 2519.247 (2107.036, 3044.259) | 10.424 (8.734, 12.551) |
| Paraguay | 528.328 (348.775, 719.450) | 8.499 (5.666, 11.602) |
| Lesotho | 107.605 (53.027, 183.304) | 7.650 (3.789, 12.867) |
| Portugal | 1618.070 (1348.744, 1944.496) | 7.229 (5.998, 8.770) |
| Somalia | 456.150 (42.848, 1310.110) | 6.209 (0.642, 17.533) |
| Taiwan (Province of China) | 2298.240 (1655.378, 3028.651) | 5.896 (4.246, 7.726) |
| Nepal | 1246.930 (328.635, 2261.361) | 5.684 (1.538, 10.122) |
| Brazil | 11748.993 (9917.173, 14178.361) | 4.820 (4.073, 5.807) |
| Eswatini | 29.615 (14.212, 50.177) | 4.628 (2.288, 7.674) |
| Italy | 7312.311 (6244.676, 8638.373) | 4.469 (3.789, 5.363) |
| Central African Republic | 106.310 (14.042, 266.159) | 4.388 (0.726, 10.607) |
| Kiribati | 2.744 (1.651, 4.466) | 4.267 (2.687, 6.445) |
| Albania | 171.043 (110.919, 262.722) | 3.994 (2.592, 6.098) |
| India | 40722.434 (21566.530, 58925.738) | 3.560 (1.908, 5.101) |
| Mexico | 4176.742 (3250.499, 5313.897) | 3.502 (2.732, 4.446) |
| Botswana | 51.966 (29.619, 85.015) | 3.420 (1.990, 5.459) |
| Namibia | 49.144 (29.497, 76.917) | 3.343 (2.026, 5.131) |
| Hungary | 599.163 (466.730, 758.315) | 3.233 (2.508, 4.119) |
| Pakistan | 3436.034 (1427.756, 5315.219) | 3.146 (1.349, 4.838) |
| South Africa | 1441.653 (1107.818, 1795.919) | 3.074 (2.387, 3.797) |
| Burundi | 144.356 (26.006, 357.582) | 3.040 (0.596, 7.365) |
| France | 4448.224 (3342.797, 6302.477) | 2.880 (2.178, 4.108) |
| Spain | 2777.516 (2217.062, 3689.109) | 2.648 (2.140, 3.473) |
| Madagascar | 303.103 (75.016, 650.899) | 2.627 (0.677, 5.507) |
| Honduras | 156.828 (54.539, 269.018) | 2.550 (0.880, 4.365) |
| Romania | 880.133 (667.952, 1133.716) | 2.531 (1.902, 3.271) |
| Mozambique | 290.376 (64.926, 624.138) | 2.509 (0.616, 5.212) |
| Democratic Republic of the Congo | 869.814 (177.566, 2182.699) | 2.378 (0.545, 5.643) |
| Bolivia (Plurinational State of) | 204.304 (113.756, 318.433) | 2.251 (1.277, 3.475) |
| Bangladesh | 2860.269 (1136.350, 4741.312) | 2.206 (0.891, 3.622) |
| Solomon Islands | 6.384 (3.342, 10.832) | 2.164 (1.090, 3.707) |
| Bulgaria | 295.639 (215.614, 408.959) | 2.131 (1.549, 2.930) |
| Slovakia | 192.681 (139.334, 259.041) | 2.105 (1.533, 2.824) |
| Eritrea | 60.377 (13.477, 139.140) | 2.047 (0.527, 4.613) |
| Japan | 8784.970 (6790.286, 12960.490) | 2.032 (1.566, 2.973) |
| Malawi | 147.510 (37.185, 320.474) | 1.972 (0.528, 4.212) |
| Bhutan | 10.273 (3.508, 18.042) | 1.906 (0.658, 3.277) |
| Papua New Guinea | 83.601 (42.508, 145.535) | 1.898 (0.901, 3.419) |
| Germany | 4305.423 (3297.345, 6432.774) | 1.886 (1.453, 2.939) |
| Colombia | 965.206 (709.885, 1274.314) | 1.849 (1.358, 2.444) |
| Rwanda | 113.394 (41.579, 211.655) | 1.843 (0.713, 3.347) |
| Uganda | 260.967 (69.687, 513.798) | 1.800 (0.516, 3.464) |
| Zimbabwe | 120.573 (79.027, 175.880) | 1.787 (1.195, 2.649) |
| Vanuatu | 2.798 (1.484, 4.767) | 1.678 (0.874, 2.810) |
| Czechia | 332.007 (234.870, 456.604) | 1.661 (1.173, 2.301) |
| Zambia | 113.132 (31.251, 222.793) | 1.639 (0.506, 3.070) |
| Slovenia | 57.986 (34.431, 95.353) | 1.582 (0.914, 2.557) |
| Monaco | 1.529 (0.855, 2.702) | 1.566 (0.881, 2.730) |
| Comoros | 7.077 (2.212, 15.001) | 1.461 (0.460, 3.032) |
| Ethiopia | 581.913 (161.569, 1248.737) | 1.420 (0.393, 2.988) |
| Marshall Islands | 0.459 (0.279, 0.705) | 1.392 (0.838, 2.125) |
| South Sudan | 51.085 (16.721, 110.426) | 1.388 (0.481, 2.947) |
| Austria | 248.318 (199.630, 320.543) | 1.383 (1.111, 1.765) |
| San Marino | 0.907 (0.540, 1.423) | 1.367 (0.807, 2.135) |
| Kenya | 304.207 (117.324, 527.424) | 1.356 (0.564, 2.280) |
| Micronesia (Federated States of) | 0.947 (0.560, 1.476) | 1.344 (0.821, 2.083) |
| United Republic of Tanzania | 322.072 (120.537, 619.070) | 1.304 (0.512, 2.430) |
| Argentina | 691.435 (543.945, 868.188) | 1.303 (1.032, 1.625) |
| Djibouti | 7.615 (3.032, 16.049) | 1.300 (0.556, 2.586) |
| Congo | 32.190 (10.705, 59.148) | 1.259 (0.475, 2.219) |
| Uruguay | 57.415 (43.611, 73.870) | 1.237 (0.963, 1.555) |
| Angola | 137.136 (50.355, 282.664) | 1.225 (0.496, 2.406) |
| Luxembourg | 12.153 (9.035, 16.104) | 1.221 (0.906, 1.620) |
| Montenegro | 11.298 (7.393, 16.306) | 1.185 (0.788, 1.696) |
| Ukraine | 846.709 (584.988, 1210.727) | 1.179 (0.810, 1.705) |
| Nauru | 0.051 (0.033, 0.075) | 1.176 (0.791, 1.651) |
| Samoa | 1.697 (1.133, 2.440) | 1.175 (0.784, 1.681) |
| Croatia | 85.838 (53.620, 135.390) | 1.171 (0.739, 1.780) |
| Haiti | 88.220 (18.504, 213.546) | 1.166 (0.230, 2.876) |
| Tuvalu | 0.114 (0.072, 0.173) | 1.165 (0.729, 1.757) |
| Serbia | 170.092 (113.859, 251.325) | 1.153 (0.778, 1.700) |
| Norway | 94.329 (66.274, 134.707) | 1.094 (0.754, 1.561) |
| North Macedonia | 34.691 (23.343, 50.324) | 1.080 (0.736, 1.574) |
| Latvia | 36.138 (23.555, 54.327) | 1.073 (0.697, 1.619) |
| Canada | 657.735 (483.118, 1310.608) | 1.031 (0.782, 1.915) |
| Tonga | 0.807 (0.511, 1.208) | 1.022 (0.647, 1.538) |
| Tokelau | 0.013 (0.008, 0.019) | 0.996 (0.652, 1.452) |
| Turkey | 895.800 (595.274, 1432.082) | 0.976 (0.652, 1.582) |
| Maldives | 3.408 (2.208, 4.970) | 0.924 (0.592, 1.374) |
| Russian Federation | 2019.362 (1373.250, 2942.255) | 0.915 (0.618, 1.331) |
| Belarus | 136.414 (85.813, 214.039) | 0.912 (0.584, 1.417) |
| Niue | 0.020 (0.013, 0.029) | 0.908 (0.594, 1.318) |
| Bosnia and Herzegovina | 50.994 (31.068, 80.394) | 0.905 (0.553, 1.417) |
| Peru | 281.463 (165.905, 503.727) | 0.870 (0.514, 1.550) |
| Gabon | 8.556 (3.423, 17.804) | 0.837 (0.360, 1.688) |
| Bahamas | 3.300 (2.466, 4.399) | 0.834 (0.627, 1.100) |
| Estonia | 18.109 (10.549, 28.423) | 0.834 (0.488, 1.328) |
| Cook Islands | 0.203 (0.125, 0.306) | 0.825 (0.510, 1.231) |
| Belgium | 195.890 (140.017, 267.632) | 0.824 (0.586, 1.136) |
| Republic of Moldova | 45.166 (25.702, 72.070) | 0.816 (0.474, 1.291) |
| Thailand | 841.778 (551.818, 1256.258) | 0.803 (0.520, 1.199) |
| Northern Mariana Islands | 0.449 (0.263, 0.716) | 0.801 (0.485, 1.254) |
| Lithuania | 38.661 (24.646, 59.684) | 0.797 (0.515, 1.200) |
| Bermuda | 0.913 (0.707, 1.165) | 0.789 (0.616, 1.000) |
| Guinea-Bissau | 9.295 (3.207, 19.500) | 0.788 (0.288, 1.520) |
| Mali | 95.296 (42.756, 179.469) | 0.785 (0.361, 1.365) |
| Timor-Leste | 6.473 (4.020, 9.861) | 0.782 (0.494, 1.188) |
| Lao People's Democratic Republic | 36.659 (22.806, 56.132) | 0.779 (0.488, 1.200) |
| Republic of Korea | 703.364 (384.327, 2284.769) | 0.779 (0.429, 2.521) |
| Myanmar | 374.907 (238.238, 554.225) | 0.770 (0.486, 1.139) |
| American Samoa | 0.375 (0.219, 0.584) | 0.767 (0.445, 1.167) |
| Equatorial Guinea | 3.491 (1.554, 7.775) | 0.763 (0.361, 1.590) |
| Ecuador | 117.419 (79.808, 181.902) | 0.757 (0.518, 1.172) |
| Guam | 1.455 (0.862, 2.299) | 0.755 (0.452, 1.183) |
| Afghanistan | 130.607 (27.910, 272.125) | 0.749 (0.177, 1.456) |
| Malaysia | 206.132 (126.098, 323.938) | 0.741 (0.455, 1.165) |
| Fiji | 5.716 (3.334, 8.856) | 0.724 (0.426, 1.119) |
| Sao Tome and Principe | 1.024 (0.458, 1.961) | 0.717 (0.346, 1.240) |
| Indonesia | 1692.208 (1115.179, 2503.949) | 0.709 (0.470, 1.049) |
| Seychelles | 0.801 (0.481, 1.268) | 0.680 (0.408, 1.070) |
| Viet Nam | 668.416 (420.012, 1038.585) | 0.670 (0.418, 1.021) |
| Armenia | 28.836 (21.822, 39.322) | 0.664 (0.505, 0.897) |
| Cambodia | 83.359 (53.033, 124.591) | 0.660 (0.425, 0.979) |
| Australia | 279.352 (186.117, 703.204) | 0.646 (0.429, 1.616) |
| Guinea | 48.154 (20.448, 87.611) | 0.641 (0.287, 1.104) |
| Mauritius | 11.471 (6.404, 18.722) | 0.639 (0.364, 1.038) |
| Yemen | 102.053 (29.059, 183.647) | 0.638 (0.183, 1.179) |
| Switzerland | 121.593 (93.895, 164.270) | 0.636 (0.495, 0.845) |
| Chad | 48.646 (19.106, 94.107) | 0.622 (0.244, 1.114) |
| Sri Lanka | 162.729 (93.859, 254.792) | 0.622 (0.359, 0.968) |
| Niger | 68.348 (22.456, 142.029) | 0.620 (0.191, 1.194) |
| Costa Rica | 32.195 (23.262, 44.081) | 0.614 (0.446, 0.837) |
| Philippines | 516.781 (337.375, 757.684) | 0.607 (0.398, 0.883) |
| Andorra | 0.803 (0.230, 3.011) | 0.570 (0.164, 2.133) |
| Iran (Islamic Republic of) | 395.504 (186.952, 496.984) | 0.537 (0.258, 0.672) |
| Sudan | 118.741 (28.660, 250.310) | 0.532 (0.130, 1.092) |
| Sierra Leone | 27.595 (10.630, 55.585) | 0.524 (0.210, 0.979) |
| Togo | 28.066 (11.671, 55.324) | 0.520 (0.226, 0.939) |
| Suriname | 3.068 (1.735, 4.851) | 0.519 (0.292, 0.809) |
| Egypt | 364.298 (96.885, 725.573) | 0.518 (0.138, 1.020) |
| Poland | 337.099 (265.748, 427.121) | 0.505 (0.399, 0.637) |
| Finland | 66.678 (35.688, 270.908) | 0.504 (0.275, 1.993) |
| Nicaragua | 20.645 (15.490, 27.419) | 0.494 (0.371, 0.653) |
| Saint Vincent and the Grenadines | 0.622 (0.453, 0.821) | 0.484 (0.352, 0.637) |
| Gambia | 6.338 (2.324, 12.920) | 0.478 (0.185, 0.892) |
| Israel | 47.299 (38.140, 60.329) | 0.473 (0.377, 0.597) |
| United States of America | 2541.497 (2130.499, 3121.595) | 0.473 (0.398, 0.580) |
| Guyana | 3.288 (2.353, 4.506) | 0.472 (0.340, 0.640) |
| Benin | 33.999 (13.918, 66.766) | 0.471 (0.211, 0.873) |
| United Kingdom | 572.532 (431.860, 860.052) | 0.469 (0.353, 0.692) |
| United Arab Emirates | 37.139 (5.734, 116.390) | 0.467 (0.108, 1.470) |
| Côte d’Ivoire | 76.070 (33.613, 158.293) | 0.450 (0.222, 0.846) |
| Cameroon | 83.426 (34.335, 170.962) | 0.443 (0.203, 0.834) |
| Netherlands | 156.672 (114.023, 270.275) | 0.432 (0.316, 0.734) |
| Senegal | 41.481 (18.835, 79.393) | 0.421 (0.201, 0.763) |
| Singapore | 29.407 (19.080, 42.941) | 0.398 (0.256, 0.585) |
| Brunei Darussalam | 0.902 (0.632, 1.272) | 0.394 (0.268, 0.567) |
| Iraq | 96.836 (51.223, 155.082) | 0.379 (0.204, 0.617) |
| Cyprus | 6.724 (4.679, 11.202) | 0.377 (0.269, 0.597) |
| Burkina Faso | 46.800 (18.429, 94.514) | 0.365 (0.151, 0.663) |
| Cabo Verde | 2.128 (1.053, 4.338) | 0.364 (0.193, 0.725) |
| New Zealand | 28.457 (16.349, 44.421) | 0.351 (0.209, 0.539) |
| Belize | 1.358 (0.847, 2.092) | 0.344 (0.227, 0.512) |
| Morocco | 103.790 (36.715, 178.149) | 0.328 (0.117, 0.552) |
| Liberia | 9.967 (3.755, 21.829) | 0.324 (0.132, 0.639) |
| Venezuela (Bolivarian Republic of) | 94.090 (68.181, 128.047) | 0.324 (0.236, 0.440) |
| Nigeria | 329.138 (200.335, 558.914) | 0.282 (0.179, 0.454) |
| Libya | 14.689 (5.287, 25.012) | 0.263 (0.101, 0.445) |
| Oman | 5.202 (2.155, 9.532) | 0.262 (0.098, 0.461) |
| Lebanon | 13.405 (5.112, 32.287) | 0.260 (0.099, 0.634) |
| Azerbaijan | 23.997 (15.689, 35.673) | 0.257 (0.170, 0.379) |
| Mauritania | 6.694 (3.480, 12.665) | 0.254 (0.145, 0.453) |
| Sweden | 53.347 (37.489, 107.734) | 0.252 (0.186, 0.462) |
| Algeria | 81.231 (36.719, 128.612) | 0.243 (0.109, 0.385) |
| Mongolia | 4.579 (2.592, 10.226) | 0.239 (0.133, 0.542) |
| El Salvador | 13.632 (9.622, 18.367) | 0.231 (0.163, 0.311) |
| Tunisia | 28.417 (11.584, 50.670) | 0.229 (0.095, 0.398) |
| Uzbekistan | 36.984 (24.692, 55.572) | 0.198 (0.135, 0.287) |
| Ghana | 36.739 (21.071, 67.158) | 0.193 (0.113, 0.327) |
| Guatemala | 21.057 (13.979, 28.896) | 0.182 (0.121, 0.252) |
| Cuba | 34.345 (25.469, 45.569) | 0.181 (0.133, 0.241) |
| Saint Lucia | 0.367 (0.266, 0.488) | 0.175 (0.127, 0.234) |
| Panama | 7.141 (4.975, 10.158) | 0.173 (0.120, 0.246) |
| Tajikistan | 8.029 (4.995, 12.122) | 0.173 (0.110, 0.259) |
| Bahrain | 1.604 (0.944, 3.201) | 0.158 (0.094, 0.361) |
| Saint Kitts and Nevis | 0.104 (0.071, 0.147) | 0.158 (0.110, 0.217) |
| Georgia | 9.678 (6.164, 14.527) | 0.157 (0.100, 0.235) |
| United States Virgin Islands | 0.245 (0.148, 0.385) | 0.156 (0.093, 0.249) |
| Grenada | 0.164 (0.121, 0.221) | 0.153 (0.114, 0.204) |
| Turkmenistan | 5.553 (3.408, 8.447) | 0.147 (0.091, 0.227) |
| Greenland | 0.096 (0.063, 0.144) | 0.144 (0.096, 0.214) |
| Kazakhstan | 23.983 (14.817, 36.505) | 0.139 (0.086, 0.214) |
| Kyrgyzstan | 6.004 (3.507, 9.419) | 0.135 (0.079, 0.212) |
| Dominica | 0.108 (0.063, 0.161) | 0.127 (0.073, 0.190) |
| Dominican Republic | 12.633 (7.478, 21.988) | 0.127 (0.076, 0.222) |
| Qatar | 0.874 (0.595, 1.302) | 0.121 (0.081, 0.182) |
| Saudi Arabia | 22.407 (14.148, 33.654) | 0.121 (0.076, 0.180) |
| Syrian Arab Republic | 12.529 (7.115, 19.268) | 0.108 (0.062, 0.162) |
| Greece | 19.718 (14.859, 25.808) | 0.094 (0.070, 0.130) |
| Ireland | 6.077 (3.241, 17.608) | 0.085 (0.046, 0.241) |
| Denmark | 9.391 (6.496, 16.964) | 0.083 (0.058, 0.151) |
| Palestine | 2.081 (1.497, 2.893) | 0.081 (0.059, 0.110) |
| Kuwait | 1.828 (1.280, 2.466) | 0.080 (0.056, 0.109) |
| Jamaica | 2.065 (1.390, 3.014) | 0.070 (0.047, 0.101) |
| Jordan | 4.125 (2.962, 5.702) | 0.062 (0.044, 0.085) |
| Barbados | 0.271 (0.203, 0.349) | 0.060 (0.045, 0.077) |
| Trinidad and Tobago | 1.032 (0.677, 1.688) | 0.060 (0.039, 0.102) |
| Antigua and Barbuda | 0.045 (0.029, 0.063) | 0.046 (0.030, 0.064) |
| Malta | 0.372 (0.234, 0.768) | 0.040 (0.025, 0.082) |
| Puerto Rico | 2.023 (1.439, 2.872) | 0.032 (0.023, 0.047) |
| Iceland | 0.128 (0.067, 0.177) | 0.023 (0.013, 0.032) |
